# Key concepts in children’s footwear research: a scoping review focusing on therapeutic footwear

**DOI:** 10.1186/s13047-019-0336-z

**Published:** 2019-04-27

**Authors:** Matthew Hill, Aoife Healy, Nachiappan Chockalingam

**Affiliations:** Centre for Biomechanics and Rehabilitation Technologies, Staffordshire University, Stoke on Trent, ST4 2DF UK

**Keywords:** Shoes, Orthotic devices, Disability, Child, Adolescent, Paediatric, Mobility limitation, Assistive devices

## Abstract

**Background:**

Reports suggest that children with mobility impairment represent a significant proportion of the population living with a disability. Footwear is considered to be the key extrinsic factor affecting children’s gait and footwear modifications have been historically postulated to assist with locomotory difficulty. Although therapeutic footwear has been considered within the literature, there is a lack of consistency on terminology and paucity on the overall understanding. A scoping review was performed to chart the key concepts in children’s footwear and to establish the range of studies that considered therapeutic footwear.

**Methods:**

A systematic search of MEDLINE, CINAHL, PubMed, SPORTdiscus, and Scopus electronic databases was performed using MeSH headings and free text terms in relation to children’s footwear. All studies that used footwear as an intervention in children aged 9 months to 18 years with the outcome measures including design, fit, and the effects on development and health were included. Studies were charted by textual narrative synthesis into research groupings dependent on the topics discussed and the methods used in the studies.

**Results:**

The search yielded a total of 5006 articles with 287 of these articles meeting the inclusion criteria. Two overarching areas of research were identified; articles that discussed footwear design and those that discussed the effects of footwear. Eight further general groupings were charted and apportioned between the overarching areas and therapeutic footwear was charted into three subgroupings (corrective, accommodative and functional).

**Conclusion:**

Children’s footwear has become an increasing area of research in the past decade with a shift towards more empirical research, with most of the included articles examining biomechanical and anthropometric aspects. However, children’s therapeutic footwear has not shared the same recent impetus with no focused review and limited research exploring its effects. Empirical research in this area is limited and there is ambiguity in the terminology used to describe therapeutic footwear. Based on the findings of this review the authors suggest the term children’s therapeutic footwear be used as the standard definition for footwear that is designed specifically with the purpose to support or alleviate mobility impairment in childhood; with subgroupings of corrective, accommodative and functional dependent on the intended therapeutic role.

**Electronic supplementary material:**

The online version of this article (10.1186/s13047-019-0336-z) contains supplementary material, which is available to authorized users.

## Background

A United Nations report on disability provided an estimate of 93 million children in the world with moderate or severe disability. This equates to 5 % of the global population under 15 years of age [1]. A further report from the United Kingdom highlighted that children represent the fastest growing group amongst the population of people with disabilities [2]. Of these childhood disabilities, over 30% are related to mobility or coordination impairment [3]. Mobility issues in children represent a significant social and health problem [3] which may require appropriate physical and rehabilitation medicine interventions to assist in their daily activities [4, 5]. Assistive devices such as orthoses, crutches and walking frames have been found to benefit individuals with mobility impairment in activities of daily living [4, 5]. Footwear is the primary interface between the individual and the ground and as such will contribute to how ground reaction forces generated in gait are applied to the foot and ankle [6]. Considering this, it is logical that footwear has been postulated to offer a role as a mobility aid for children with locomotory impairment since the eighteenth Century [4, 7–9]. Research has shown that footwear is the key extrinsic factor affecting children’s gait with studies on conventional footwear in healthy children demonstrating that it modifies: lower limb movements, forces and sensory stimulus acting through the foot [6, 10–13]. As children are still growing and developing their feet demonstrate differing structural and functional characteristics in comparison to adult feet [14–16]. These differences will also vary within childhood depending on the developmental stage taking into account the: plasticity of the foot, growth rate, allometry, and motor ability [17–19]. It is therefore considered that foot development is a fundamental factor underlying the requirements of children’s footwear [6, 20]. However, there is still uncertainty on the long-term effects of footwear on child development and the specifics of children’s footwear design in terms of support and flexibility [6, 20, 21]. These uncertainties concerning footwear are further confounded when considering the developmental needs of children living with a physical disability [20, 22, 23].

Therapeutic footwear for children consists of a number of footwear modifications that may be either bespoke or off-the-shelf [23, 24]. These modifications have been used in an attempt to achieve efficient walking patterns or to correct skeletal alignment in children with a range of clinical presentations such as: flat feet, talipes equino varus, toe walking, cerebral palsy, and developmental delay [23, 25–27]. Footwear intended for therapeutic use ranges in design and application from those whose role is to simply accommodate a foot orthosis to those that act as an independent mobility or corrective device [8, 24, 28]. Therapeutic footwear is widely prescribed by healthcare professionals, as evidenced by a recent survey in the United Kingdom [29]; however, there is lack of scientific evidence on the specifics of the design and purpose of this footwear for children.

Conventional children’s footwear in typically developing children has been examined in a number of reviews, including the effects of footwear on gait and the requirements of athletic and school footwear [6, 17, 30]. Although children’s therapeutic footwear has previously been considered in a number of reviews, some of these have focussed on individual pathological conditions and others have provided an overview rather than a structured synthesis of the body of research [31–33]. Therefore, it is important to establish the range, and scope of research focussing on therapeutic footwear to support future evidence base in this area. However, it is unclear how footwear intended for therapeutic purposes in children has been defined in the literature. Thus, in order to identify the scope of work concerning therapeutic footwear it is first essential to establish the terminology used for this intervention within the general body of children’s footwear research.

A systematic search was undertaken to compile the key concepts pertaining to children’s footwear that is facilitative of daily wear, and activity to demonstrate the volume, and progress of work in this area. It was also performed to highlight the gaps in knowledge whilst considering therapeutic footwear alongside the body of children’s footwear research. In addition, it was important to include all areas of research and not just limit to either the design and manufacturing aspects of footwear or their influence on locomotory function.

The review set out to achieve the following objectives:

1. Explore how children’s footwear has been studied in the literature; specifically, the intended purpose of the footwear and the chosen methodology.
2. Identify how therapeutic footwear has been defined and studied in terms of its design and intended therapeutic role.

With the overall aim to summate the current state and scope of knowledge in relation to both conventional and therapeutic children’s footwear and to inform further research streams on the role of footwear as a therapy for children with locomotory disability.

## Methods

The scoping review followed the staged methodological guidance of Arksey & O’Malley [34] and Colquhoun et al. [35] this met the preferred reporting guidelines extension for scoping reviews PRISMA-ScR [36] (see Additional file 1 for PRISMA-ScR Checklist). The inclusion and exclusion criteria for the systematic search is detailed below.

### Inclusion and exclusion criteria

#### Types of study and publication

##### Inclusion

- Studies where footwear was the intervention or where its effects were explored independently if it was used as an adjunct to an orthotic intervention.
- Studies examining characteristics relating to ergonomic footwear design and fit.
- Studies exploring the effects of footwear on child health
- Studies exploring the effects of footwear on child development.
- All study designs were considered from peer-reviewed journals and conference proceedings
- Studies with an available English language abstract.

##### Exclusion

- Studies where footwear was not the preliminary or secondary focus of the research question.
- Commercial based study design customisation which was not related to fit or function.
- Textbook entries, poster presentations.
- Non-English language abstract.

#### Participants

##### Inclusion

- Infant, children, and adolescents of typical walking and shod age 9 months-18 years of age.

##### Exclusion

- Less than 9 months of age.
- Greater than 18 years of age.

#### Footwear type

##### *Inclusion*

- Footwear that facilitates typical daily activities (e.g., walking running, jumping)

##### *Exclusion*

- Footwear modified for specific sporting task precluding daily wear and activities (e.g. studs, cleats, spikes, ski-boots, and skates)

### Search strategy

The following electronic databases were searched for eligible studies: MEDLINE, CINAHL, PubMed, SPORTdiscus, and Scopus. MeSH headings and free text terms for children and footwear were used to capture all research in this area. Search strategy including the search terms is provided in supplementary material (Additional file 2). The search strategy was adapted across the databases to capture eligible articles published from database inception to 1st February 2018.

### Screening and selection of studies

Prior to screening all duplicates were removed using referencing software (Mendeley, Elsevier B.V.) and supplemented by a manual check by the principal investigator (PI). One reviewer (PI) independently screened the titles and abstracts yielded by the search against the eligibility criteria; with any uncertainty regarding eligibility resolved through discussion with the 2nd and 3rd reviewers.

### Data extraction and synthesis

Data were extracted from the abstracts by the PI using a form developed and tested by the PI. Information on study design, footwear style, age range of participants, methodology, outcomes of the study and topics discussed were extracted. Textual narrative synthesis [37, 38] was used to chart the evidence into sectioned homogeneous research groupings dependent on the topics discussed or the methodology used within the studies. The charting process took an iterative approach, with groupings of the research reached by structured discussion and consensus between all reviewers. As data was extracted solely from the abstracts, i.e. the full texts of the included studies were not analysed, a quality assessment of the included studies were not performed.

## Results

**Fig. 1:**
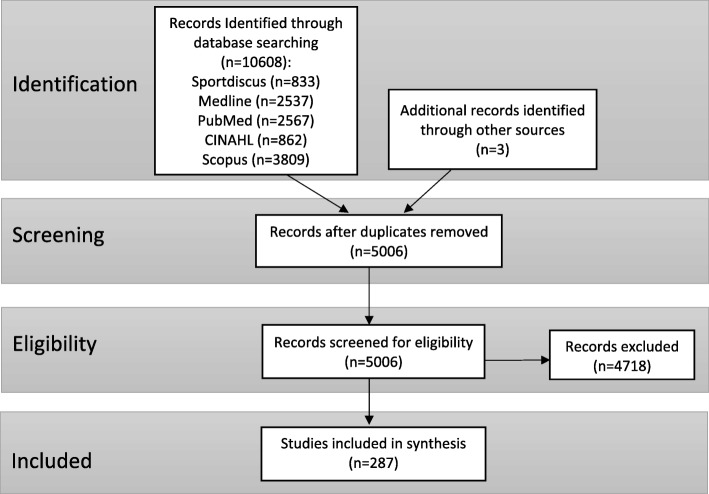
Flow diagram for selection of studies included in the scoping review

The search yielded 10,608 articles, after removing duplicate articles this total was reduced to 5003 articles. Three further articles were found through related author research [39–41]. Following screening, a total of 287 articles were included for synthesis (Fig. 1). A full list of the included studies and results of individual sources of evidence are provided in Additional file 3 (n = number of papers from included studies).

**Fig. 2:**
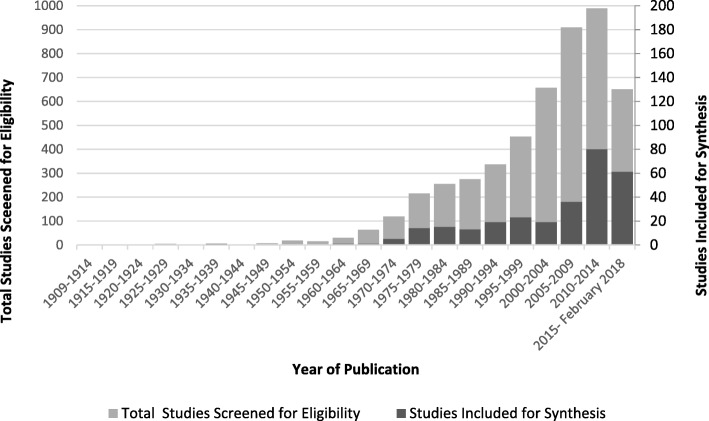
Scoping review search results by year of publication

When articles were grouped by year of publication (Fig. 2), it was evident that children’s footwear is an increasing area of research with 56% of articles (*n* = 161) identified in this search published in the past 10 years. There were 211 empirical studies amongst the articles sourced, with 137 of these reporting the age range of the participants in the abstract; age range was from 9 months to 18 years. Articles were grouped by age into 3 ranges: 1) **infant and preschool** (9mths-5Yrs), 2) **primary school** (6-12Yrs) and 3) **adolescents** (13-18Yrs). Although a number of articles considered more than one of the age groupings in the population sampled the majority of the research involved primary school aged children (*n* = 93), followed by adolescents (*n* = 56), then infants and pre-schoolers (*n* = 53).

Charting of the included articles yielded two overarching areas of research in children’s footwear:

1. **Footwear design** (*n* = 146) this was in terms of both ergonomics (refining the dimensional fit and functional properties of footwear to meet the daily demands of the child’s foot in both typical and atypical development) and the material components of footwear (upper, lining, sole and tanning agents).
2. The **effects of footwear** (*n* = 216) on the child (effects on gait, protective benefits, risk factor for injury/pathology and therapeutic effects).

**Fig. 3:**
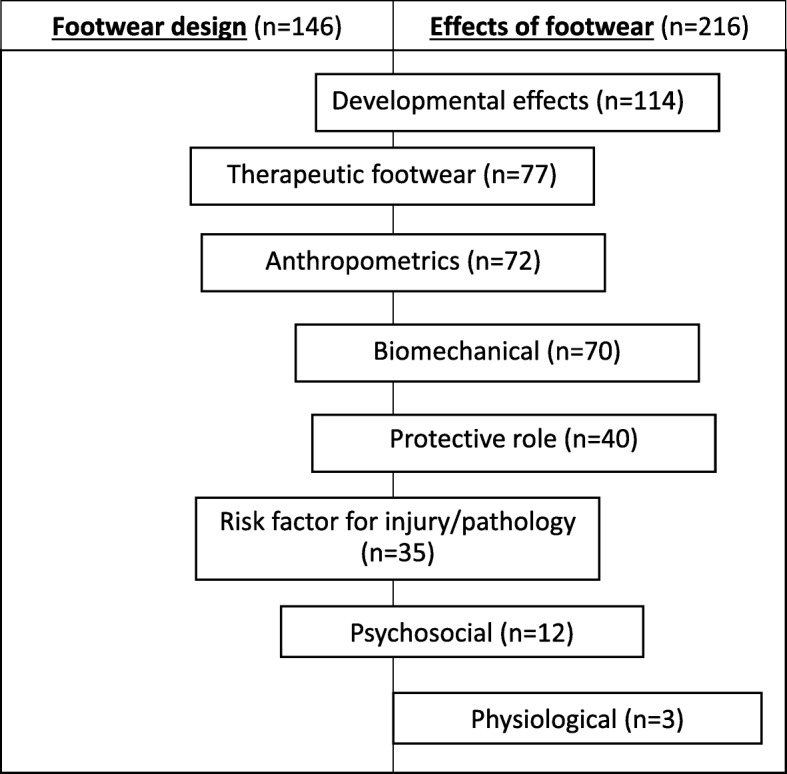
Charting of studies within overarching areas (footwear design and effects) and groupings (ordered by volume of studies)

Amongst these two overarching areas, eight general groupings were further charted. Figure 3 provides an overview of the charted groupings and how the articles in each group were apportioned amongst the two main areas. The articles were not exclusive to each of the eight groupings or two overarching areas with many articles overlapping across both areas and groupings.

### Developmental effects

These articles explored the effects or perceived effects of footwear and footwear design on typical and atypical child development; this represented the largest research grouping (*n* = 114). Ninety-four of the studies were empirical in design with age range reported in 63 of the articles, infant and preschool (*n* = 28), primary school (*n* = 40) and adolescents (*n* = 19). Earlier research in this grouping focused on skeletal foot development (*n* = 35) inclusive of the medial longitudinal arch and digital deformity [42–47]. However the recent focus of this research grouping has considered the potential effects on neuromuscular development in terms of gait and other motor tasks (*n* = 45) [6, 25, 48–52]. The remaining articles (*n* = 35) were in relation to the ideal attributes of footwear design and application for the child in both typical and atypical development, with a broad range study design including opinion base, cross-sectional survey through to systematic review [22, 53–55].

### Therapeutic footwear

This grouping focused on footwear that was designed for the treatment of childhood musculoskeletal or neurological locomotor disability with the underlying principle of last and sole modification to influence the structure and function of the child’s foot [8, 23, 24, 33, 56–58]. Numerous terms were used to define therapeutic footwear throughout the literature including orthopaedic shoes, shoe corrections, rehabilitative boots, modified shoes, arch support footwear, supportive shoes, special shoes, medical shoes and wedged shoes [23, 25, 56, 59–64]. Of the 77 articles in this group, 23 explored the effects of therapeutic footwear empirically with the age range given in 9 of these articles; age groups were roughly equally represented in these studies: infant and preschool (*n* = 6), primary school (*n* = 7) and adolescent (*n* = 5).

**Fig. 4:**
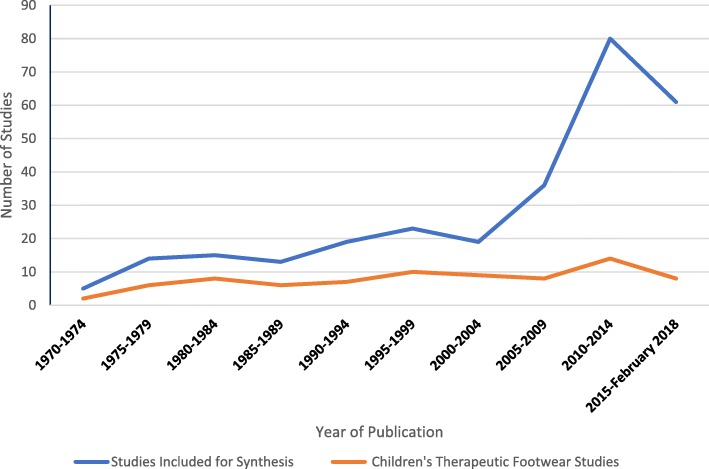
Volume of children’s therapeutic footwear articles compared to the total volume of children’s footwear articles published annually

Figure 4 compares the number of therapeutic footwear articles by year of publication to the total articles considered for synthesis in this review. Although the volume of articles on children’s therapeutic footwear has increased annually since the 1970s, when compared to the total volume of research in children’s footwear its proportion of this total volume has decreased; from 35% of the total articles from 1998 to 2007 to 17% of the total articles from 2008 to 2018.

Therapeutic footwear was charted, based on the information provided within the abstract, into three separate subgroupings (corrective, accommodative, and functional) according to the perceived therapeutic role of the footwear. Of the 77 articles, 38 were related to corrective footwear, 34 functional, 2 to accommodative, 5 articles did not specify the direct clinical aims or outcomes of the footwear. One paper discussed corrective, functional and accommodative therapeutic footwear [28]).

#### Corrective footwear

Corrective footwear was defined in this review as footwear that was designed to bring about correction of congenital skeletal lower limb alignment [8, 65]. Corrective footwear research yielded several footwear design modifications that were used to treat a range of structural lower limb issues (e.g., Talipes Equino Varus, genu varum, genu valgum, tibial torsion, paediatric pes planus, metatarsus adductus and hallux valgus) [27, 43, 56, 66–68]. The types of footwear included Thomas heel, high topped, reverse last, straight last, in-built arch support, reinforced steel shank, and loop sandals [7, 43, 56, 62, 68].

The effects of corrective footwear have been mainly assessed by prospective studies (n = 11) examining anthropometric measures of the medial longitudinal arch including radiographic, laser scanning, and footprint analysis [24, 27, 43, 56, 62, 69–73]. Other articles (*n* = 13) included expert opinion on corrective footwear in terms of design and conditions treated [59, 74–76], review articles (*n* = 7) [31, 33, 67, 77], psychosocial considerations (*n* = 4) [78, 79] and clinical prescription surveys (*n* = 3) [7, 69].

#### Accommodative footwear

This was defined within this review as footwear that was designed (modular or bespoke) to reduce compression and shearing stresses on the child’s foot deformity through dimensional matching of footwear upper, insole, and sole to that of the child’s foot [28, 80]. There was a dearth of research (*n* = 2) in terms of children’s accommodative therapeutic footwear [28, 80]. Of the two articles, one was an opinion piece on suggested indications for therapeutic footwear in terms of “misshapen feet” [28] the second article sourced was a review where accommodative footwear was considered as part of the suggested management for digital deformity in childhood [80].

#### Functional footwear

This was defined as footwear designed to improve dynamic gait parameters of children with mobility impairment, reducing pathological movements and facilitating typical childhood walking patterns [4, 25]. Functional therapeutic footwear consisted of four further subgroupings which were charted dependent on design and the perceived functional role: stability (*n* = 25), lift (*n* = 8), rounded bottom sole (*n* = 1) and instability (*n* = 1).

Stability therapeutic footwear is a range of footwear that is designed to limit extreme movements of the lower limb in order to maintain a controlled displacement of the centre of force during gait [23, 28]. Various footwear designs (toplines that extend above malleoli, stiffened extended heel counters, stiffened sole, wedged sole, and torqheel) [28, 61, 64, 81] have been used to impart stability and these may be used in isolation or in combination with ankle-foot orthosis tuning [58]. The range of childhood mobility disorders where they have been used includes: cerebral palsy, muscular dystrophy, toe walking, in-toeing, spina bifida, pes planus, haemophilic arthropathy and developmental delay [4, 5, 25, 61, 82–84].

Research on the effects of stability therapeutic footwear, on body posture and gait, was limited (*n* = 9) but has included case studies through to cross-sectional study of anthropometrics and biomechanical parameters [23, 25, 61, 64, 85–87]. Other articles included opinion based pieces (*n* = 8) on the design and clinical use of stability footwear [28, 60, 81, 88], review articles (*n* = 7) [4, 5, 32, 58, 89] and a survey of their use in muscular dystrophy (*n* = 1) [82].

Lift therapeutic footwear was defined as a unilateral modular footwear sole addition to conservatively achieve postural and functional symmetry in individuals with limb length inequality [90], this included both functional and structural limb length difference of 1 cm or greater found in such conditions as cerebral palsy and idiopathic scoliosis [9, 91]. The effects of lift therapeutic footwear have been reported (n = 4) in relation to spinal posture, objective gait parameters and symptomatic relief [9, 91–93]. Other articles were opinion based with respect to clinical indications and the degree of lift required [28, 94, 95].

The effect of rounded bottom therapeutic footwear on gait was studied in one conference proceeding abstract [96]. This footwear consists of a sole with a forefoot rocker design proposed to assist sagittal plane progression of the foot and toe clearance in stiff knee gait associated with cerebral palsy.

Instability therapeutic footwear consists of a sole designed to promote imbalance with the intention of training the individuals motor coordination. The effects on static and reactive balance and directional control in children with developmental delay were assessed in one pilot study [97].

### Anthropometrics

This grouping of articles was in reference to the methods employed in the research which involved the objective study of the human body in relation to dimension, geometry and proportions. The majority of articles (*n* = 66) were of foot measures (length, width, height, circumference, toe flex angle); however the effects of heel height on spinal posture was also reported in the literature (*n* = 6) [91, 98]. Methods involved direct measurement of anatomy or measurements from imaging modalities; these included callipers, inked and pressure foot-printing, radiological imaging, and 3D dynamic laser scanning [42, 99, 100]. Forty-four of the abstracts reported the age range in these studies with the age groups represented in the following number of articles, infant and preschool (*n* = 20), primary school (*n* = 29), and adolescents (*n* = 22).

The anthropometric grouping of articles were distributed into articles of footwear design (*n* = 36) which related anthropometric data to ergonomic design of children’s footwear taking into consideration the age and perceived rate of foot growth [18, 101–106], gender [106], geographic region [107], body type [108], and developmental pathology [47]. The other articles (*n* = 2) considered the use of anthropometrics to formulate footwear assessment scores to quantify footwear fit in children [55, 109]. A considerable number of articles (*n* = 34) used anthropometric methods to study the immediate or potential long term consequence of footwear on children’s anatomy, including the medial longitudinal arch, forefoot width, digital deformity, and lumbar lordosis [46, 110–112].

### Biomechanical

Like the anthropometrics grouping, this grouping was in relation to the methods used in the research. These studies involved the mechanical effects of footwear on the child’s locomotory system, including gait (running, walking), and motor tasks (jumping, balance) [48, 113–115]. These studies utilised, kinetic, kinematic, electromyography, and spatio-temporal assessments [6, 48, 49, 116]. Footwear designs studied included “*school footwear*,” athletic footwear, therapeutic footwear, and thong style flip-flops [6, 25, 117].

A focus on biomechanics involving children’s footwear has been an increasing area of research with a total of 55 of the included 70 articles published in the past 10 years. Fifty of the abstracts reported the age range in these studies: infant and preschool (*n* = 15), primary school (*n* = 38), and adolescents (*n* = 16). Biomechanical studies have chiefly been used to assess the potential effects of footwear on both typical and atypical motor development (*n* = 46) [49, 118]. Other studies assessed the short term biomechanical effects of footwear (*n* = 6) [23, 114], the potential biomechanical design requirements of footwear (e.g., fastenings, fit, heel height, and upper and sole material stiffness) (*n* = 15) [12, 119–122], or explored footwear as a secondary experimental variable to orthotic intervention (*n* = 3) [123–125].

### Protective role

The research in this grouping studied the role of children’s footwear in reducing the risk of injury or pathology. This was divided into three subgroupings: **1)** infection articles (*n* = 30) examining the reduction of childhood parasitic disease in developing countries [126–129], **2)** environmental articles (*n* = 4) exploring the prevention of lacerations, puncture wounds, and environmental irritants [130, 131] and **3)** functional articles (n = 6) examining the potential of footwear to reduce injury or pain through increased traction, stability, and cushioning [132–135].

### Risk factor for injury/pathology

This grouping considered the role of footwear as a potential cause of injury or pathology. This was divided into three subgroupings: **1)** dermatology (*n* = 23) these articles focused on the material properties of footwear leading to reactive skin pathologies [136–138]. **2)** injury (n = 7) these articles discussed features such as design, fit or “ageing” of the footwear, that increases the likelihood of trauma from activity or the environment [139–141]. **3)** infection articles (*n* = 5) which examined the effect of the material properties of footwear in creating an internal environment of the footwear that is conducive to increased risk of microbial infection [142, 143].

### Psychosocial

This grouping involved articles that discussed and studied personal or parental beliefs of footwear design in terms of child development, protective function, and social identity. Parents were surveyed (*n* = 6) on their views and understanding of footwear and potential effects on foot development [79, 144–146]. Adolescents were surveyed (*n* = 2) on what influenced their selection of athletic footwear [147, 148]. Concerning social identity (*n* = 4) the effect of the type or design of footwear on self-image, self-esteem, and social isolation were examined [149–152].

### Physiological

These articles (*n* = 3) compared the cardiovascular, respiratory, and metabolic effects between shod and unshod walking and running in children [153–155]. Parameters studied included the Physiological Cost Index (PCI), oxygen consumption and calorific cost. Both children with typical development and cerebral palsy have been amongst the populations studied [154, 155]. This was the only research grouping where there was no apparent discussion or comparison of footwear design within the articles.

## Discussion

This current scoping review demonstrated that children’s footwear in general is an increasing area of research with most of the articles in this area published within the past 10 years. It has also highlighted the range of research evidence has developed from opinion base, to more objective and structured research methodologies.

In consideration of the two overarching areas, footwear design and effects of footwear**,** the articles tended to discuss and study the effects of footwear on the child; however, there was a sizable number of articles (*n* = 70) that considered footwear design in terms of the fit of the footwear. Footwear fit relates to the ergonomic purpose of footwear, a significant factor of its function is how it fits the foot [156]. Even though fit appeared to be a prominent area of research, there was a limited number of empirical studies (n = 4) exploring the effects of incorrectly fitted footwear on children [46, 47, 122, 157].

The protective role of footwear was considered in a number of articles; however, this has chiefly been in relation to reduced risk of parasitic infection with only a limited number of articles exploring protection from physical sources.

Growth and development are a defining characteristic of childhood consequently developmental effects of footwear were noted to be the largest of the general research groupings in the sourced literature (*n* = 114). Consistent with the overall trend of research in children’s footwear 65% of the total articles from this grouping were published in the past 10 years and there has been a shift in the studies from opinion base towards empirical research, with this now representing 78% of the available literature in this research grouping.

The methods used in children’s footwear research both in their design and to explore their effects on the child mainly consisted of biomechanical and anthropometric studies, with a minority of studies considering the physiological and psychosocial effects. In consideration of typical development a number of biomechanical studies now exist which compare barefoot and shod conditions on children’s gait and other motor tasks [6, 12, 49, 158, 159]. The majority of these biomechanical studies were carried out in children of primary school age compared to the other age groupings.

In consideration of atypical development both foot deformity and neuromuscular conditions have a demonstrable effect on a child’s daily activity [4, 160]. Since footwear is the primary interface between the foot and the ground these conditions may require specific footwear needs in relation to fit and function [4], with footwear having the potential to act as a therapeutic aid to assist locomotion in childhood disability [123, 161].

Therapeutic Footwear appears to have been well documented in the literature but in contrast to the trend of research in children’s footwear, which has increased substantially in recent years, less than a third of the articles were produced in the past ten years (Fig. 4). The majority of this research is based on dated opinion [28, 75, 83, 162] with empirical studies on the effects of therapeutic footwear limited to 30% of the available research [23–25, 70].

There are numerous terms, design, and therapeutic roles attributed to footwear in the literature and this scoping review attempted to form groupings and consistent terminology to structure this research area. The narrative charting of the articles suggested the terminology of children’s therapeutic footwear to cover all aspects of children’s footwear that is designed with the specific purpose to assist mobility impairment in childhood. With therapeutic footwear being divided into the subgroupings of corrective, accommodative and functional dependent on the perceived role of the footwear. This may potentially offer clarity to further research and clinical usage in this area.

Amongst the subgroupings of therapeutic footwear corrective and functional footwear were the most studied. The emphasis of recent research in children’s therapeutic footwear appears to be shifting towards a functional intervention on children’s walking rather than correction of foot postures such as pes planus, however, articles in these subgroupings still demonstrated a relatively low volume of studies compared to the total volume of recent children’s footwear research. The literature in relation to children’s therapeutic footwear appears to show a number of gaps in knowledge in terms of empirical study of its effects, the definition and design of this footwear and clear guidelines for their use as a therapeutic intervention.

It is considered best practice to manage healthcare conditions holistically in terms of physiological, psychological, and sociological consideration [4, 163]. The International Classification of Functioning, Disability and Health-Children and Youth version devised by the World Health Organisation [164] provides a logical framework to assess how a child’s condition and environment may allow or restrict them to function in a multitude of everyday activities. Further research which objectively establishes the effects of therapeutic footwear in terms of body function and daily activities are needed to support the development of guidelines for clinical populations which would benefit from footwear interventions. This approach will allow children with mobility impairment to achieve their fullest level of function and participation in daily life, whilst avoiding prescription of interventions that may be of little effect, reducing unnecessary healthcare costs and potential psychosocial detriment to the child [24, 69, 79, 152].

Although this review has fulfilled its objectives in order to define and categorise children’s therapeutic footwear and showcase the progress of the work in this area the limitations of the current study are recognised. Whilst agreement of the research groupings and included studies were met through consensus amongst the reviewers, the exclusion of studies and data extraction was performed independently by the PI, which may have opened these processes to personal bias. In addition, this review has considered only those articles with an available English language abstract which may have impacted on the scope of research.

## Conclusion

This scoping review has established that children’s footwear has become an increasing area of research in the past decade. Although therapeutic footwear has been discussed in a considerable number of articles it has represented a smaller proportion of the recent research into children’s footwear.

The articles were narratively grouped into eight general groups with the overarching areas of footwear design and footwear effects; most of the articles examined the biomechanical and anthropometric aspects of footwear. However, in relation to children’s therapeutic footwear, there is still limited empirical research in children and ambiguity in the terminology used to define this type of footwear.

To offer potential clarity to future research in this area; this scoping review suggests the term children’s therapeutic footwear be used as the common definition for footwear that is designed specifically with the purpose to support or alleviate locomotor disability in childhood. With the sub groupings of corrective, accommodative, and functional to be applied dependent on the intended therapeutic role of the footwear. A further focused systematic review is required to establish the quality of evidence in relation to therapeutic footwear and inform future research streams.

## Additional files

Additional file 1: File format: Preferred Reporting Items for Systematic reviews and Meta-Analyses extension for Scoping Reviews (PRISMA-ScR) Checklist. Description of data: Checklist of the scoping review process for the current study against recommended guidelines. (DOCX 107 kb)

Additional file 2: Example of Medline (EBSCO) search strategy. Description of data: Medline database search strategy inclusive of Free text, MeSH and Boolean terms. (DOCX 12 kb)

Additional file 3: Results of individual sources of evidence. Description of data: Full alphabetical listing by author of all included studies in the current scoping review and the charted data characteristics for each study. (DOC 365 kb)
