# Supplementary material for: Key concepts in children’s footwear research: a scoping review focusing on therapeutic footwear
Source: J Foot Ankle Res. 2019 Apr 27;12:25. doi: 10.1186/s13047-019-0336-z (PMC6487054; doi:10.1186/s13047-019-0336-z)
Supplement: Supplementary file 2 — Example of Medline (EBSCO) search strategy. Description of data: Medline database search strategy inclusive of Free text, MeSH and Boolean terms. (DOCX 12 kb) [file 13047_2019_336_MOESM2_ESM.docx]

**Additional File 1**

Example of Medline (EBSCO) search strategy

1. Child*.ti,ab.

2. Infant*ti,ab.

3. Adolescent *ti,ab.

4. Paediatric*ti,ab.

5. Pediatric*ti,ab.

6. Schoolchild*ti,ab.

7. Toddler*ti,ab.

8. Preschool*ti,ab.

9. teenage*ti,ab.

10. Exp Child/

11. Exp Infant/

12. Adolescent/

13. 1 OR 2 OR 3 OR 4 OR 5 OR 6 OR 7 OR 8 OR 9 OR 10 OR 11 OR 12

14. Shoe*.ti,ab.

15. Footwear*.ti,ab.

16. Boot.ti,ab.

17. Boots.ti,ab.

18. Sandal*.ti,ab.

19. Exp Shoes/

20. 14 OR 15 OR 16 OR 17 OR 18 OR 19

21. 13 AND 20
