# Supplementary material for: Key concepts in children’s footwear research: a scoping review focusing on therapeutic footwear
Source: J Foot Ankle Res. 2019 Apr 27;12:25. doi: 10.1186/s13047-019-0336-z (PMC6487054; doi:10.1186/s13047-019-0336-z)
Supplement: Supplementary file 3 — Results of individual sources of evidence. Description of data: Full alphabetical listing by author of all included studies in the current scoping review and the charted data characteristics for each study. (DOC 365 kb) [file 13047_2019_336_MOESM3_ESM.doc]

**Additional File 3 Results of individual sources of evidence.**

| **Article** | **Study Design** | **Age/Age Range** | **Charted:**  **Area/ Grouping/ Sub Grouping** |
| --- | --- | --- | --- |
| 1. Abd Elkader, S. M., Abd Elhafz, Y. N. & Al-Abdulrazaq, S. S. Foot taping versus medical shoes on kinematic gait parameters in children with down’s syndrome. World Appl. Sci. J. 27, 311–317 (2013). | Randomised Control Trial | Not Reported in Abstract | Biomechanics, Developmental Effects, Effects of Footwear, Therapeutic Footwear, Therapeutic Footwear Functional, Therapeutic-Footwear Stability |
| 2. Abera**,** B., Alem, G., Yimer, M. & Herrador, Z. Epidemiology of soil-transmitted helminths, Schistosoma mansoni, and haematocrit values among schoolchildren in Ethiopia. J. Infect. Dev. Ctries. 7, 253–260 (2013). | Cross Sectional Study | Primary School 6-12yrs | Effects of Footwear, Protective Role, Protective Role Infective |
| 3. Abolarin, T., Aiyegbusi, A., Tella, A. & Akinbo, S. Predictive factors for flatfoot: The role of age and footwear in children in urban and rural communities in South West Nigeria. Foot 21, 188–192 (2011). | Cross Sectional Study | Primary School 6-12yrs | Anthropometrics, Developmental Effects, Effects of Footwear |
| 4. Aboutorabi, A. et al. Immediate effect of orthopedic shoe and functional foot orthosis on center of pressure displacement and gait parameters in juvenile flexible flat foot. Prosthet Orthot Int 38, 218–223 (2014). | Before-and-after study | Not Reported in Abstract | Biomechanics, Effects of Footwear, Therapeutic Footwear, Therapeutic Footwear Functional, Therapeutic Footwear Functional Stability |
| 5. Adams, D. W. & Marshall-Battle, M. R. Shoe contact dermatitis: a case report of an acute severe reaction to potassium dichromate. Foot 22, 141–145 (2012). | Case Study | Not Reported in Abstract | Risk Factor Injury/Pathology, Risk Factor Dermatology, Effects of Footwear, Footwear Design |
| 6. Aibast, H. et al. Foot Structure and Function in Habitually Barefoot and Shod Adolescents in Kenya. Curr. Sport. Med. Reports (Lippincott Williams Wilkins) 16, 448–458 (2017). | Cross Sectional Study | Adolescent 13-18yrs, Primary School 6-12yrs | Anthropometrics, Biomechanics, Developmental Effects, Effects of Footwear |
| 7. Aimpun, P. & Hshieh, P. Survey for intestinal parasites in Belize, Central America. Southeast Asian J Trop Med Public Heal. 35, 506–511 (2004). | Cross Sectional Study | Not Reported in Abstract | Effects of Footwear, Protective Role,  Protective Role Infective |
| 8. Akiko, Y. A Study of the Foot Form for Footwear Design. Part 4: The Application of Principal Component Analysis to the Property of Foot Form of Children Aged 3 to 6. J. JAPAN Res. Assoc. Text. END-USES 31, 533–538 (1990). | Cross Sectional Study | Preschool and Infants 9mths-5yrs | Anthropometrics, Footwear Design |
| 9. Al-Delaimy, A. K. et al. Epidemiology of intestinal polyparasitism among Orang Asli school children in rural Malaysia. PLoS Negl Trop Dis 8, e3074–e3074 (2014). | Cross Sectional Study | Not Reported in Abstract | Effects of Footwear, Protective Role, Protective Role Infective |
| 10. Alelign, T., Degarege, A. & Erko, B. Soil-Transmitted Helminth Infections and Associated Risk Factors among Schoolchildren in Durbete Town, Northwestern Ethiopia. J. Parasitol. Res. 2015, 641602 (2015). | Cross Sectional Study | Not Reported in Abstract | Effects of Footwear, Protective Role, Protective Role Infective |
| 11. Alemu, A. et al. Soil transmitted helminths and schistosoma mansoni infections among school children in zarima town, northwest Ethiopia. BMC Infect. Dis. 11, 189 (2011). | Cross Sectional Study | Not Reported in Abstract | Effects of Footwear, Protective Role, Protective Role Infective |
| 12. Alemu, A., Tegegne, Y., Damte, D. & Melku, M. Schistosoma mansoni and soil-transmitted helminths among Preschool-aged children in Chuahit, Dembia district, Northwest Ethiopia: prevalence, intensity of infection and associated risk factors. BMC Public Health 16, 1–9 (2016). | Cross Sectional Study | Preschool and Infants 9mths-5yrs | Effects of Footwear, Protective Role, Protective Role Infective |
| 13. Als, C. & Marugg, S. Exclusive wearing of shoes of impregnated cloth by an adolescent girl during a cold winter: Late effects in osseous tomoscintigraphy and in magnetic resonance imaging. Med. Nucl. 33, 658–661 (2009). | Case Study | 14 years | Effects of Footwear, Footwear Design, Risk Factor Injury/Pathology, Risk Factor Injury |
| 14. Au, I. P. H. et al. Immediate and short-term biomechanical adaptation of habitual barefoot runners who start shod running. J Sport. Sci 36, 451–455 (2018). | Randomised Control Trial | Adolescent 13-18yrs | Biomechanics, Effects of Footwear |
| 15. Ayala, F. et al. A multicentre study of contact sensitization in children. Gruppo Italiano Ricerca Dermatiti da Contatto e Ambientali (GIRDCA). Contact Dermatitis 26, 307–310 (1992). | Cross Sectional Study | Not Reported in Abstract | Risk Factor Injury/Pathology, Risk Factor Dermatology, Effects of Footwear, Footwear Design |
| 16. Ayode, D. et al. A Qualitative Study Exploring Barriers Related to Use of Footwear in Rural Highland Ethiopia: Implications for Neglected Tropical Disease Control. PLoS Negl Trop Dis 7, e2199–e2199 (2013). | Survey | Not Reported in Abstract | Protective Role, Psychosocial, Effects of Footwear, Protective Role Infective |
| 17. Baba, K. Shoes as a necessity for children. J Hum Ergol 5, 82–83 (1976). | Opinion Piece | Not Applicable | Developmental Effects, Footwear Design |
| 18. Bailey-Van Kuren, M., Gillette, S., Mejia, P., Stoever, T. & Walker, A. Design considerations for a wearable pediatric rehabilitative boot. in 2005 IEEE 9th International Conference on Rehabilitation Robotics, ICORR 2005 2005, 400–403 (2005). | Opinion Piece | Not Applicable | Therapeutic Footwear, Therapeutic Footwear Functional, Therapeutic Footwear Functional Stability, Footwear Design |
| 19. Baker, M. D. & Bell, R. E. The role of footwear in childhood injuries. Pediatr Emerg Care 7, 353–355 (1991). | Cross Sectional Study | Not Reported in Abstract | Protective Role, Protective Role Functional. Risk Factor Injury/Pathology, Risk Factor Injury, Footwear Design, Effects of Footwear |
| 20. Bakker, J. P. J., De Groot, I. J. M., De Jong, B. A., Van Tol-De Jager, M. A. & Lankhorst, G. J. Prescription pattern for orthoses in The Netherlands: use and experience in the ambulatory phase of Duchenne muscular dystrophy. Disabil. Rehabil. 19, 318–325 (1997). | Survey | Not Reported in Abstract | Therapeutic Footwear, Therapeutic Footwear Functional, Therapeutic Footwear Functional Stability, Footwear Design, Effects of Footwear |
| 21. Bari, S. B., Othman, M. & Mohd Salleh, N. Foot anthropometry for shoe design among Preschool children in Malaysia. Pertanika J. Soc. Sci. Humanit. 18, 69–79 (2010). | Cross Sectional Study | Preschool and Infants 9mths-5yrs | Anthropometrics, Footwear Design |
| 22. Barisch-Fritz, B., Plank, C. & Grau, S. Evaluation of the rule-of-thumb: calculation of the toe allowance for developing feet. Footwear Sci. 8, 119–127 (2016). | Cross Sectional Study | Adolescent 13-18yrs, Primary School 6-12yrs | Anthropometrics, Footwear Design |
| 23. Barisch-Fritz, B., Schmeltzpfenning, T., Plank, C., Hein, T. & Grau, S. The effects of gender, age, and body mass on dynamic foot shape and foot deformation in children and adolescents. Footwear Sci. 6, 27–39 (2014). | Cross Sectional Study | Adolescent 13-18yrs, Primary School 6-12yrs | Anthropometrics, Footwear Design |
| 24. Barisch-Fritz, B., Schmeltzpfenning, T., Plank, C. & Grau, S. Foot deformation during walking: Differences between static and dynamic 3D foot morphology in developing feet. Ergonomics 57, 921–933 (2014). | Cross Sectional Study | Adolescent 13-18yrs, Primary School 6-12yrs | Anthropometrics, Footwear Design |
| 25. Bartkowlak, Z. et al. Orthopedic equipment applied in children with cerebral palsy. Fizjoterapia / Physiother. 16, 99–113 (2008). | Narrative Review | Not Reported in Abstract | Therapeutic Footwear, Therapeutic Footwear Functional, Therapeutic Footwear Functional Stability, Effects of Footwear |
| 26. Basta, N. W. et al. A comparative study of the role of shoes, arch supports, and navicular cookies in the management of symptomatic mobile flat feet in children. Int. Orthop. 1, 143–148 (1977). | Cohort Study | Not Reported in Abstract | Anthropometrics, Developmental Effects, Therapeutic Footwear, Therapeutic Footwear Corrective, Footwear Design, Effects of Footwear |
| 27. Beattie, P. E., Green, C., Lowe, G. & Lewis-Jones, M. S. Which children should we patch test? Clin Exp Dermatol 32, 6–11 (2007). | Case Series | Adolescent 13-18yrs, Preschool and Infants 9mths-5yrs, Primary School 6-12yrs | Effects of Footwear, Risk Factor Injury/Pathology, Risk Factor Dermatology, Footwear Design |
| 28. Becerril-Chihu, G. et al. How often are dermatophytes present in apparently normal versus scaly feet of children? Pediatr Dermatol 16, 87–89 (1999). | Cross Sectional Study | Preschool and Infants 9mths-5yrs, Primary School 6-12yrs | Risk Factor Injury/Pathology, Risk Factor Infective, Effects of Footwear, Footwear Design |
| 29. Benbella, I. et al. [Cutaneous larva migrans syndrome on a malformed foot (a case report)]. Pan Afr. Med. J. 23, 50 (2016). | Case Study | 15 months | Effects of Footwear, Protective Role,  Protective Role Infective |
| 30. Berger, C. Children’s shoe: a miniature version of the adult shoe? Sport. Sport. 7, 183–186 (1993). | Opinion Piece | Not Applicable | Developmental Effects, Footwear Design |
| 31. Bernardczyk, K. The influence of the footwear on the health status of the child’s foot. Chir Narzadow Ruchu Ortop Pol 38, 233–238 (1973). | Opinion Piece | Not Applicable | Developmental Effects, Footwear Design |
| 32. Bernhard, M. K. & Merkenschlager, A. Does barefoot walking influence the prevalence of idiopathic toe walking? Differences between German and Bengal children. Padiatr. Prax. 72, 301–305 (2008). | Cross Sectional Study | Preschool and Infants 9mths-5yrs, Primary School 6-12yrs | Biomechanics, Developmental Effects, Effects of Footwear |
| 33. Bhaskara Rao, U., Joseph, B., Rao, U. B. & Joseph, B. The influence of footwear on the prevalence of flat foot. A survey of 2300 children. J Bone Jt. Surg Br 74, 525–527 (1992). | Cross Sectional Study | Primary School 6-12yrs | Anthropometrics, Developmental Effects, Effects of Footwear, Footwear Design |
| 34. Bird, C., Ame, S., Albonico, M. & Bickle, Q. Do shoes reduce hookworm infection in school-aged children on Pemba Island, Zanzibar? A pragmatic trial. Trans R Soc Trop Med Hyg 108, 297–304 (2014). | Randomised Control Trial | Not Reported in Abstract | Effects of Footwear, Protective Role, Protective Role Infective |
| 35. Bleck, E. E. The Shoeing of Children: Sham or Science? Dev. Med. Child Neurol. 13, 188–195 (1971). | Cross Sectional Study | Not Reported in Abstract | Anthropometrics, Developmental Effects, Therapeutic Footwear, Therapeutic Footwear Corrective, Effects of Footwear, Footwear Design |
| 36. Blitz, J. R., Stern, S. & Marzan, K. A. B. A110: The Impact of Shoe Wear on the 6 Minute Walk Test in Adolescents with Juvenile Idiopathic Arthritis. Arthritis Rheumatol. 66, S146–S146 (2014). | Pilot Study | Adolescent 13-18yrs | Biomechanics, Effects of Footwear, Protective Role, Protective Role Functional, Footwear Design, |
| 37. Böhm, H. et al. Effect of floor reaction ankle-foot orthosis on crouch gait in patients with cerebral palsy: What can be expected? Prosthet Orthot Int 42, 309364617716240–309364617716240 (2017). | Before-and-after study | Adolescent 13-18yrs, Primary School 6-12yrs | Biomechanics, Effects of Footwear |
| 38. Bordelon, R. L. Hypermobile flatfoot in children: present status of diagnosis and treatment. Semin. Orthop. 5, 13–22 (1990). | Opinion Piece | Not Applicable | Therapeutic Footwear, Therapeutic Footwear Corrective, Developmental Effects, Effects of Footwear |
| 39. Bordelon, R. L. Hypermobile flatfoot in children. Comprehension, evaluation, and treatment. Clin Orthop Relat Res NO. 181, 7–14 (1983). | Opinion Piece | Not Applicable | Therapeutic Footwear, Therapeutic Footwear Corrective, Effects of Footwear, Developmental Effects |
| 40. Branthwaite, H., Chockalingam, N., Grogan, S. & Jones, M. Footwear choices made by young women and their potential impact on foot health. J Heal. Psychol 18, 1422–1431 (2013). | Survey | Adolescent 13-18yrs | Psychosocial, Effects of Footwear, Footwear Design |
| 41. Buckland, M. A. et al. The Effect of Torsional Shoe Flexibility on Gait and Stability in Children Learning to Walk. Pediatr Phys Ther 26, 417 (2014). | Before-and-after study | Preschool and Infants 9mths-5yrs | Biomechanics, Developmental Effects, Effects of Footwear, Footwear Design |
| 42. Butler, P. et al. Physiological cost index of walking for normal children and its use as an indicator of physical handicap. Dev Med Child Neurol 26, 607–612 (1984). | Before-and-after study | Preschool and Infants 9mths-5yrs, Primary School 6-12yrs | Biomechanics, Physiological, Effects of Footwear |
| 43. Byrne, M. et al. The development and use of a footwear assessment score in comparing the fit of children’s shoes. Foot 8, 215–218 (1998). | Cross Sectional Study | Not Reported in Abstract | Anthropometrics, Developmental Effects, Effects of Footwear, Footwear Design |
| 44. Camper, P. The classic: Dissertation on the best form of shoe, Pieter Camper. Clin Orthop Relat Res No. 110, 2–5 (1975). | Opinion Piece | Not Reported in Abstract | Therapeutic Footwear, Footwear Design |
| 45. Carstensen, H. & Baumann, J. U. Orthopedic care of the feet in patients with cerebral disorders of motion. Ther. Umschau 31, 18–22 (1974). | Opinion Piece | Not Applicable | Therapeutic Footwear, Therapeutic Footwear Functional, Therapeutic Footwear Functional Stability, Footwear Design |
| 46. Caselli, M. A., Rzonca, E. C. & Lue, B. Y. Habitual toe-walking: evaluation and approach to treatment. Clin Pod. Med Surg 5, 547–559 (1988). | Opinion Piece | Not Applicable | Therapeutic Footwear, Therapeutic Footwear Functional, Therapeutic Footwear Functional Stability, Effects of Footwear |
| 47. Chard, A., Greene, A., Hunt, A., Vanwanseele, B. & Smith, R. Effect of thong style flip-flops on children’s barefoot walking and jogging kinematics. J Foot Ankle Res 6, 8 (2013). | Before-and-after study | Primary School 6-12yrs | Biomechanics, Effects of Footwear, Developmental Effects, Footwear Design |
| 48. Chen, J.-P. P., Chung, M.-J. J., Wu, C.-Y. Y., Cheng, K.-W. W. & Wang, M.-J. J. Comparison of Barefoot Walking and Shod Walking Between Children with and Without Flat Feet. J Am Pod. Med Assoc 105, 218–225 (2015). | Before-and-after study | Primary School 6-12yrs | Biomechanics, Effects of Footwear, Developmental Effects |
| 49. Chen, W. et al. Correcting Congenital Talipes Equinovarus in Children Using Three Different Corrective Methods: A Consort Study. Med. (United States) 94, e1004–e1004 (2015). | Cross Sectional Study | Not Reported in Abstract | Anthropometrics, Developmental Effects, Therapeutic Footwear, Therapeutic Footwear Corrective, Biomechanics, Effects of Footwear, Footwear Design |
| 50. Choy, S. H. et al. Prevalence and associated risk factors of Giardia infection among indigenous communities in rural Malaysia. Sci Rep 4, 6909 (2014). | Cross Sectional Study | Not Reported in Abstract | Effects of Footwear, Protective Role,  Protective Role Infective |
| 51. Cockayne, S. E., Shah, M., Messenger, A. G. & Gawkrodger, D. J. Foot dermatitis in children: causative allergens and follow-up. Contact Dermatitis 38, 203–206 (1998). | Cross Sectional Study | Not Reported in Abstract | Effects of Footwear, Risk Factor Injury/Pathology, Risk Factor Dermatology, Footwear Design |
| 52. Coll Bosch, M. D., Viladot Perice, A. & Suso Vergara, A. Follow-up study of flat feet in children. Rev. Ortop. y Traumatol. 43, 213–220 (1999). | Non Randomised Control Trial | Preschool and Infants 9mths-5yrs | Anthropometrics, Biomechanics, Effects of Footwear, Developmental Effects, Therapeutic Footwear, Therapeutic Footwear Corrective |
| 53. Colloud, F. et al. Shoes effect on young children gait with the increase of displacement velocity. Mov. Sport. Sci. - Sci. Mot. 75, 97–105 (2012). | Before-and-after study | Not Reported in Abstract | Biomechanics, Effects of Footwear, Developmental Effects, Footwear Design |
| 54. Coughlin, M. J. Juvenile Hallux Valgus: Etiology and Treatment. Foot Ankle Int 16, 682–697 (1995). | Cross Sectional Study | Not Reported in Abstract | Anthropometrics, Developmental Effects, Footwear Design, Effects of Footwear |
| 55. Cowell, H. R. Shoes and shoe corrections. Pediatr Clin North Am 24, 791–797 (1977). | Opinion Piece | Not Applicable | Therapeutic Footwear, Therapeutic, Developmental effects, Footwear Corrective, Footwear Design |
| 56. Da Rocha, E. S., Bratz, D. T. K., Gubert, L. C., De David, A. & Carpes, F. P. Obese children experience higher plantar pressure and lower foot sensitivity than non-obese. Clin. Biomech. 29, 822–827 (2014). | Cross Sectional Study | Not Reported in Abstract | Biomechanics, Effects of Footwear, Developmental Effects, Footwear Design |
| 57. Dai, M. et al. High-heeled-related alterations in the static sagittal profile of the spino-pelvic structure in young women. Eur Spine J 24, 1274–1281 (2015). | Before-and-after study | Not Reported in Abstract | Anthropometrics, Footwear Design, Developmental Effects, Effects of Footwear |
| 58. Davies, N., Branthwaite, H. & Chockalingam, N. Where should a school shoe provide flexibility and support for the asymptomatic 6- to 10-year-olds and on what information is this based? A Delphi yielded consensus. Prosthet. Orthot. Int. 39, 213–8 (2015). | Survey | Primary School 6-12yrs | Footwear Design, Developmental Effects, |
| 59. Davis, I. What Can We Learn from Watching Children Run? AMAA J. 24, 7–8 (2011). | Opinion Piece | Not Applicable | Footwear Design, Developmental Effects |
| 60. de Oliveira Pezzan, P. A. & de Freitas Lopes, D. M. in Posture: Types, Exercises and Health Effects 171–191 (Nova Science Publishers, Inc., 2014). | Cross Sectional Study | Adolescent 13-18yrs | Anthropometrics, Developmental Effects, Footwear Design, Effects of Footwear |
| 61. de Oliveira Pezzan, P. A. et al. Postural assessment of lumbar lordosis and pelvic alignment angles in adolescent users and nonusers of high-heeled shoes. J Manip. Physiol Ther 34, 614–621 (2011). | Cross Sectional Study | Adolescent 13-18yrs | Anthropometrics, Developmental Effects, Footwear Design, Effects of Footwear |
| 62. De Vetten, A. L. & Heesters, P. J. J. The qualities of children’s shoes (Dutch). Ned Tijdschr Geneeskd 120, 2044–2049 (1976). | Opinion Piece | Not Reported in Abstract | Footwear Design, Developmental Effects |
| 63. Delgado-Abellán, L. et al. Foot morphology in Spanish school children according to sex and age. Ergonomics 57, 787–797 (2014). | Cross Sectional Survey | Primary School 6-12yrs | Anthropometrics, Footwear Design |
| 64. Desloovere, K. et al. How can push-off be preserved during use of an ankle foot orthosis in children with hemiplegia? A prospective controlled study. Gait Posture 24, 142–151 (2006). | Before-and-after study | Primary School 6-12yrs | Biomechanics, Effects of Footwear |
| 65. Desmons, F. Contact dermatitis in children. Allergol Immunopathol (Madr) 3, 35–42 (1975). | Case Series | Not Reported in Abstract | Effects of Footwear, Risk Factor Injury/Pathology, Risk Factor Dermatology, Footwear Design |
| 66. Dias, M. P. et al. IDENTIFICAÃÃO DOS FATORES DE RISCO PARA ACIDENTES NA PRIMEIRA INFÃNCIA NO CONTEXTO CRECHE. Rev. Atencao Primaria a Saude 16, 20–26 (2013). | Cross Sectional Study | Preschool and Infants 9mths-5yrs | Effects of Footwear, Risk Factor Injury/Pathology, Risk Factor Injury, Footwear Design |
| 67. Dohi, M. & Koike, M. Consumers’ awareness concerning the selection of infant shoes. J. JAPAN Res. Assoc. Text. END-USES 41, 39–48 (2000). | Survey | Preschool and Infants 9mths-5yrs | Psychosocial, Footwear Design, Developmental Effects |
| 68. Driano, A. N., Staheli, L. T. & Staheli, L. R. The psychosocial effects of childhood corrective shoe wearing. J. Investig. Med. 44, 101A (1996). | Cross Sectional Study | Not Reported in Abstract | Psychosocial, Therapeutic Footwear, Therapeutic Footwear Corrective, Footwear Design, Effects of Footwear |
| 69. Driano, A. N., Staheli, L. T. & Staheli, L. T. Psychosocial development and corrective shoewear use in childhood. J. Pediatr. Orthop. 18, 346–349 (1998). | Cross Sectional Study | Not Reported in Abstract | Psychosocial, Therapeutic Footwear, Therapeutic Footwear Corrective, Footwear Design, Effects of Footwear |
| 70. Echarri, J. J. & Forriol, F. Development of footprint morphology in congolese children in relation to footwear use. Rev. Ortop. y Traumatol. 47, 395–399 (2003). | Cross Sectional Study | Preschool and Infants 9mths-5yrs, Primary School 6-12yrs | Anthropometrics, Developmental Effects, Effects of Footwear, Cross Sectional Study |
| 71. Echarri, J. J. & Forriol, F. The development in footprint morphology in 1851 Congolese children from urban and rural areas, and the relationship between this and wearing shoes. J. Pediatr. Orthop. Part B 12, 141–146 (2003). | Cross Sectional Study | Preschool and Infants 9mths-5yrs, Primary School 6-12yrs | Anthropometrics, Developmental Effects, Effects of Footwear, Cross Sectional Study |
| 72. Eddison, N. & Chockalingam, N. The effect of tuning ankle foot orthoses-footwear combination on the gait parameters of children with cerebral palsy. Prosthet. Orthot. Int. 37, 95–107 (2013). | Narrative Review | Not Reported in Abstract | Therapeutic Footwear, Therapeutic Footwear Functional, Therapeutic Footwear Functional Stability, Effects of Footwear |
| 73. Eddison, N., Healy, A., Needham, R. & Chockalingam, N. Shank-to-Vertical Angle in Ankle-Foot Orthoses: A Comparison of Static and Dynamic Assessment in a Series of Cases. J. Prosthetics Orthot. 29, 161–167 (2017). | Case Series | Not Reported in Abstract | Biomechanics, Effects of Footwear, ,Therapeutic Footwear, Therapeutic Footwear Functional, Therapeutic Footwear Functional Stability |
| 74. Eek, M. N., Zügner, R., Stefansdottir, I. & Tranberg, R. Kinematic gait pattern in children with cerebral palsy and leg length discrepancy: Effects of an extra sole. Gait Posture 55, 150–156 (2017). | Before-and-after study | Adolescent 13-18yrs, Primary School 6-12yrs | Biomechanics, Effects of Footwear, Therapeutic Footwear, Therapeutic Footwear Functional, Therapeutic Footwear Functional Lift |
| 75. Eiff, M. P., Steiner, E., Judkins, D. Z. & Winkler-Prins, V. Clinical inquiries. What is the appropriate evaluation and treatment of children who are ‘toe walkers’? J Fam Pr. 55, 447 (2006). | Narrative Review | Not Reported in Abstract | Therapeutic Footwear, Therapeutic Footwear Functional, Therapeutic Footwear Functional Stability, Effects of Footwear |
| 76. Elyana, F. N. et al. A tale of two communities: intestinal polyparasitism among Orang Asli and Malay communities in rural Terengganu, Malaysia. Parasites and Vectors 9, 398 (2016). | Cross Sectional Study | Not Reported in Abstract | Effects of Footwear, Protective Role,  Protective Role Infective |
| 77. English, M. P., Gibson, M. D. & Duncan, E. H. L. Studies in the epidemiology of tinea pedis. Br Med J 2, 573–576 (1960). | Cross Sectional Study | Not Reported in Abstract | Effects of Footwear, Risk Factor Injury/Pathology, Risk Factor Infective, Footwear Design |
| 78. Enke, R. C., Laskowski, E. R. & Thomsen, K. M. Running shoe selection criteria among adolescent cross-country runners. PM R J. Inj. Funct. Rehabil. 1, 816–819 (2009). | Survey | Adolescent 13-18yrs | Psychosocial, Footwear Design |
| 79. Espinosa-Muñoz, D. Y., Gómez-Gómez, N. E., Polanco, L. C., Cardona-Arias, J. A. & Ríos-Osorio, L. A. Prevalence of gastrointestinal parasite in the indigenous community of Seminke of Wiwa shelter, in Sierra Nevada de Santa Marta, 2014. Arch. Med. 11, (2015). | Cross Sectional Study | Adolescent 13-18yrs, Preschool and Infants 9mths-5yrs, Primary School 6-12yrs | Effects of Footwear, Protective Role, Protective Role Infective |
| 80. Evans, A. M. & Rome, K. A Cochrane review of the evidence for non-surgical interventions for flexible pediatric flat feet. Eur. J. Phys. Rehabil. Med. 47, 69–89 (2011). | Systematic Review | Preschool and Infants 9mths-5yrs, Primary School 6-12yrs | Therapeutic Footwear, Therapeutic Footwear Corrective, Effects of Footwear  Developmental Effects |
| 81. Flores, J. M., Castillo, V. B., Franco, F. C. & Huata, A. B. Superficial fungal infections: Clinical and epidemiological study in adolescents from marginal districts of Lima and Callao, Peru. J. Infect. Dev. Ctries. 3, 313–317 (2009). | Cross Sectional Study | Adolescent 13-18yrs | Effects of Footwear, Risk Factor Injury/Pathology, Risk Factor Infective, Footwear Design |
| 82. Foiasi, T. & Pantazi, M. Children’s footwear - Health, comfort, fashion. Leather Footwear J. 10, 45–60 (2010). | Opinion Piece | Not Applicable | Footwear Design, Developmental Effects |
| 83. Fong, D. T.-P. P. et al. Cushioning and lateral stability functions of cloth sport shoes. Sport. Biomech 6, 407–417 (2007). | Before-and-after study | Primary School 6-12yrs | Biomechanics, Effects of Footwear, Footwear Design, Protective Role, Protective Role Functional |
| 84. Forrest, D., Dufek, J. S. & Mercer, J. A. Impact Characteristics of Female Children Running in Adult Versus Youth Shoes of the Same Size. J Appl Biomech 28, 593–598 (2012). | Before-and-after study | Primary School 6-12yrs | Biomechanics, Effects of Footwear, Footwear Design |
| 85. Freeman, S. Shoe dermatitis. Australas J Dermatol 24, 63–68 (1983). | Technical Report | Not Applicable | Effects of Footwear, Risk Factor Injury/Pathology, Risk Factor Dermatology, |
| 86. Frigerio, S. et al. Knowledge, Attitudes, and Practices Related to Schistosomiasis Among Children in Northern Senegal. Ann. Glob. Heal. 82, 840–847 (2016). | Cross Sectional Study | Not Reported in Abstract | Effects of Footwear, Protective Role, Protective Role Infective |
| 87. Fritz, B., Schmeltzpfenning, T., Plank, C. & Grau, S. Development of well-fitting shoes for children and adolescents. Footwear Sci. 5, S93–S94 (2013). | Cross Sectional Study | Adolescent 13-18yrs, Primary School 6-12yrs | Anthropometrics, Footwear Design |
| 88. Ganesh, M. S. P. & Magnani, B. The Influence of Footwear on the Prevalence of Flat Foot. Indian J. Physiother. Occup. Ther. 10, 157–159 (2016). | Cross Sectional Study | Primary School 6-12yrs, | Anthropometrics, Developmental Effects, Effects of Footwear |
| 89. Ganjehie, S., Saeedi, H., Farahmand, B. & Curran, S. The efficiency of gait plate insole for children with in-toeing gait due to femoral antetorsion. Prosthetics Orthot. Int. (Sage Publ. Ltd.) 41, 51–57 (2017). | Before-and-after study | Primary School 6-12yrs | Biomechanics, Effects of Footwear, Developmental Effects, Effects of Footwear |
| 90. García-Rodríguez, A. et al. Flexible flat feet in children: a real problem? Pediatrics 103, 1278 (1999). | Cross Sectional Study | Preschool and Infants 9mths-5yrs, Primary School 6-12yrs | Anthropometrics, Developmental Effects, Therapeutic Footwear, Therapeutic Footwear Corrective, Effects of Footwear |
| 91. George, D. A. & Elchert, L. The influence of foot orthoses on the function of a child with developmental delay. Pediatr. Phys. Ther. 19, 332–336 (2007). | Case Study | 19 months | Biomechanics, Effects of Footwear, Developmental Effects |
| 92. Gilmore, A. & Thompson, G. H. Common childhood foot deformities: to treat, to wait, or to refer? Many are benign and self-correcting, but parental concerns must be addressed. J. Musculoskelet. Med. 19, 287–295 (2002). | Opinion Piece | Not Applicable | Therapeutic Footwear, Therapeutic Footwear Corrective, Effects of Footwear  Developmental Effects |
| 93. Gould, N. Shoes versus sneakers in toddler ambulation. Foot Ankle 6, 105–107 (1985). | Cross Sectional Study | Preschool and Infants 9mths-5yrs | Biomechanics, Effects of Footwear, Developmental Effects, Footwear Design |
| 94. Gould, N., Moreland, M., Alvarez, R., Trevino, S. & Fenwick, J. Development of the child’s arch. Foot Ankle 9, 241–245 (1989). | Randomised Control Trial | Preschool and Infants 9mths-5yrs | Anthropometrics, Developmental Effects, Therapeutic Footwear, Therapeutic Footwear Corrective, Footwear Design, Effects of Footwear |
| 95. Gould, N. et al. Foot growth in children age one to five years. Foot Ankle 10, 211–213 (1990). | Before-and-after study | Preschool and Infants 9mths-5yrs | Preschool and Infants 9mths-5yrs, Anthropometrics, Footwear Design |
| 96. Grieve, D. W. & Gear, R. J. The relationships between length of stride, step frequency, time of swing and speed of walking for children and adults. Ergonomics 9, 379–399 (1966). | Before-and-after study | Adolescent 13-18yrs, Preschool and Infants 9mths-5yrs, Primary School 6-12yrs | Biomechanics, Effects of Footwear, Developmental Effects |
| 97. Grueger, B., Leung, A., Otley, A., Society, C. P. & Committee, G. Footwear in children. Paediatr. Child Health 14, 121–122 (2009). | Narrative Review | Not Reported in Abstract | Footwear Design, Developmental Effects, Therapeutic Footwear, Therapeutic Footwear Corrective |
| 98. Hafez, E. Effect of rounded bottom profile shoes on foot clearance in children with stiff knee gait. Gait Posture 57, 239–240 (2017). | Pilot Study | Not Reported in Abstract | Biomechanics, Effects of Footwear, Therapeutic Footwear, Therapeutic Footwear Functional, Therapeutic Footwear Functional Rounded-Bottom-Sole, Footwear Design |
| 99. Hailegebriel, T. Prevalence of intestinal parasitic infections and associated risk factors among students at Dona Berber primary school, Bahir Dar, Ethiopia. BMC Infect. Dis. 17, 1–8 (2017). | Cross Sectional Study | Primary School 6-12yrs | Effects of Footwear, Protective Role, Protective Role Infective |
| 100. Helfand, A. E. Basic considerations for shoes, shoe modifications, and orthoses in foot care. Clin Pod. 1, 431–440 (1984). | Opinion Piece | Not Applicable | Footwear Design, Therapeutic Footwear |
| 101. Herbaut, A. et al. Influence of minimalist shoes on lower-limb overuse injuries risk in children. Sci. Sport. 32, 119–128 (2017). | Narrative Review | Adolescent 13-18yrs, Primary School 6-12yrs | Effects of Footwear, Protective Role, Protective Role Functional, Footwear Design |
| 102. Herbaut, A. et al. The influence of shoe aging on children running biomechanics. Gait Posture 56, 123–128 (2017). | Before-and-after study | Not Reported in Abstract | Biomechanics, Effects of Footwear, Risk Factor Injury/Pathology, Risk Factor Injury |
| 103. Herbaut, A. et al. Determination of optimal shoe fitting for children tennis players: Effects of inner-shoe volume and upper stiffness. Appl Erg. (2017). doi:10.1016/j.apergo.2017.05.016 | Before-and-after study | Primary School 6-12yrs | Biomechanics, Effects of Footwear, Footwear Design |
| 104. Herkt, R. The Forest Town boot as an aid in treatment of cerebral palsy. Krankengymnastik 27, 367–368 (1975). | Opinion Piece | Not Applicable | Therapeutic Footwear, Therapeutic Footwear Functional, Therapeutic Footwear Functional Stability, Footwear Design |
| 105. Herro, E. & Jacob, S. E. p-tert-Butylphenol formaldehyde resin and its impact on children. Dermatitis 23, 86–88 (2012). | Technical Report | Not Reported in Abstract | Effects of Footwear, Risk Factor Injury/Pathology, Risk Factor Dermatology, Footwear Design |
| 106. Hettigama, I. S., Punchihewa, H. K. G. & Heenkenda, N. K. Ergonomic footwear for Sri Lankan primary schoolchildren: A review of the literature. Work 55, 285–295 (2016). | Narrative Review | Primary School 6-12yrs | Footwear Design, Developmental Effects |
| 107. Hicks, J. F. FITTING A POPULATION OF FEET. J. Test. Eval. 16, 404–406 (1988). | Cross Sectional Study | Not Reported in Abstract | Anthropometrics, Footwear Design |
| 108. Hillstrom, H. et al. Torsional shoe flexibility effects on functional performance of children learning to walk. Footwear Sci. 1, 73–75 (2009). | Before-and-after study | Preschool and Infants 9mths-5yrs | Biomechanics, Effects of Footwear, Developmental Effects, Footwear Design |
| 109. Hillstrom, H. J. et al. Effect of shoe flexibility on plantar loading in children learning to walk. J Am Pod. Med Assoc 103, 297–305 (2013). | Before-and-after study | Preschool and Infants 9mths-5yrs | Biomechanics, Effects of Footwear, Developmental Effects, Footwear Design |
| 110. Hobbs, S. A., Altman, K. & Halldin, M. A. Modification of a child’s deviant walking pattern: An alternative to surgery. J. Behav. Ther. Exp. Psychiatry 11, 227–229 (1980). | Case Study | 3.5 years | Therapeutic Footwear, Therapeutic Footwear Functional, Therapeutic Footwear Functional Stability, Effects of Footwear, Footwear Design |
| 111. Hollander, K. et al. Growing-up (habitually) barefoot influences the development of foot and arch morphology in children and adolescents. Sci Rep 7, 8079 (2017). | Cross Sectional Study | Not Reported in Abstract | Anthropometrics, Developmental Effects, Effects of Footwear |
| 112. Hollander, K. et al. Foot Strike Patterns Differ Between Children and Adolescents Growing up Barefoot vs. Shod. Int. J. Sports Med. 39, 97–103 (2018). | Cross Sectional Study | Adolescent 13-18yrs, Primary School 6-12yrs | Biomechanics, Effects of Footwear, Developmental Effects |
| 113. Hollander, K., Riebe, D., Campe, S., Braumann, K.-M. M. & Zech, A. Effects of footwear on treadmill running biomechanics in preadolescent children. Gait Posture 40, 381–385 (2014). | Before-and-after study | Adolescent 13-18yrs, Primary School 6-12yrs | Biomechanics, Effects of Footwear, Developmental Effects |
| 114. Hollander, K. et al. The effects of being habitually barefoot on foot mechanics and motor performance in children and adolescents aged 6-18 years: study protocol for a multicenter cross-sectional study (Barefoot LIFE project). J Foot Ankle Res 9, 36 (2016). | Research Protocol | Adolescent 13-18yrs, Primary School 6-12yrs | Developmental Effects, Effects of Footwear |
| 115. Holt, K. S. Mobility aids and appliances for disabled children. Bmj 302, 105–107 (1991). | Opinion Piece | Not Applicable | Therapeutic Footwear, Therapeutic Footwear Corrective, Therapeutic Footwear Functional, Therapeutic Footwear Functional Lift, Therapeutic Footwear Functional Stability, Footwear Design, Therapeutic Accommodative, Developmental effects |
| 116. Hon, K. L. E. et al. Does age or gender influence quality of life in children with atopic dermatitis? Clin Exp Dermatol 33, 705–709 (2008). | Cross Sectional Study | Adolescent 13-18yrs, Primary School 6-12yrs | Effects of Footwear, Risk Factor Injury/Pathology, Risk Factor Dermatology, Pathology |
| 117. Hulstaert, E. et al. Contact dermatitis caused by a new rubber compound detected in canvas shoes. Contact Dermatitis 78, 12–17 (2018). | Cross Sectional Study | Adolescent 13-18yrs | Effects of Footwear, Risk Factor Injury/Pathology, Risk Factor Dermatology, Footwear Design |
| 118. Hutchinson, B. Pediatric metatarsus adductus and skewfoot deformity. Clin Pod. Med Surg 27, 93–104 (2010). | Opinion Piece | Not Applicable | Therapeutic Footwear, Therapeutic Footwear Corrective, Effects of Footwear,  Developmental Effects |
| 119. Ilechukwu, G. C. et al. Some behavioural risk factors for intestinal helminthiasis in nursery and primary school children in Enugu, south eastern Nigeria. Niger. J. Clin. Pract. 13, 288–293 (2010). | Cross Sectional Study | Preschool and Infants 9mths-5yrs, Primary School 6-12yrs | Effects of Footwear, Protective Role, Protective Role Infective |
| 120. Imaizumi, K., Akimoto, M., Kobayashi, Y., Hobara, H. & Kouchi, M. Effects of oversized footwear on gait parameters in children. Footwear Sci. 7, S16–S18 (2015). | Before-and-after study | Primary School 6-12yrs | Biomechanics, Effects of Footwear, Footwear Design, Developmental Effects |
| 121. Ivanyi, B. et al. The effects of orthoses, footwear, and walking aids on the walking ability of children and adolescents with spina bifida: A systematic review using International Classification of Functioning, Disability and Health for Children and Youth (ICF-CY) as a ref. Prosthet Orthot Int 39, 437–443 (2015). | Systematic Review | Not Reported in Abstract | Therapeutic Footwear, Therapeutic Footwear Functional, Therapeutic Footwear Functional Stability, Effects of Footwear |
| 122. Jafarnezhadgero, A., Majlesi, M. & Madadi-Shad, M. The effects of low arched feet on lower limb joints moment asymmetry during gait in children: A cross sectional study. Foot 34, 63–68 (2018). | Cross Sectional Study | Not Reported in Abstract | Biomechanics, Effects of Footwear, Footwear Design |
| 123. James, A. M., Williams, C. M. & Haines, T. P. Effectiveness of footwear and foot orthoses for calcaneal apophysitis: a 12-month factorial randomised trial. Br J Sport. Med 50, 1268–1275 (2016) | Randomised Control Trial | Adolescent 13-18yrs, Primary School 6-12yrs | Effects of Footwear, Protective Role, Protective Role Functional |
| 124. Jane MacKenzie, A., Rome, K. & Evans, A. M. The efficacy of nonsurgical interventions for pediatric flexible flat foot: A critical review. J. Pediatr. Orthop. 32, 830–834 (2012). | Systematic Review | Not Reported in Abstract | Therapeutic Footwear, Therapeutic Footwear Corrective, Effects of Footwear,  Developmental Effects |
| 125. Jelić, M. Orthopedic devices for children. Paediatr. Croat. Suppl. 44, 205–210 (2000). | Opinion Piece | Not Reported in Abstract | Therapeutic Footwear, Footwear Design |
| 126. Jiménez-Ormeño, E. et al. Foot morphology in normal-weight, overweight, and obese schoolchildren. Eur. J. Pediatr. 172, 645–652 (2013). | Cross Sectional Study | Primary School 6-12yrs | Anthropometrics, Footwear Design |
| 127. João, S. M. A., Cardillo, C., Kieling, I., de Oliveira Pezzan, P. A. & Sauer, J. F. Analysis of the medial longitudinal arch in adolescents user of high heeled shoes. Fisioter. e Pesqui. 19, 20–25 (2012). | Cross Sectional Study | Adolescent 13-18yrs | Anthropometrics, Developmental Effects, Effects of Footwear, Footwear Design |
| 128. Joffe, M., Torrey, S. B. & Baker, M. D. Fire hydrant play: injuries and their prevention. Pediatrics 87, 900–903 (1991). | Cross Sectional Study | Not Reported in Abstract | Effects of Footwear, Protective Role, Protective Role Environmental |
| 129. Jones, S. K., English, J. S. C., Forsyth, A. & Mackie, R. M. Juvenile Plantar Dermatosis—an 8‐year follow‐up of 102 patients. Clin Exp Dermatol 12, 5–7 (1987). | Cohort Study | Not Reported in Abstract | Effects of Footwear, Risk Factor Injury/Pathology, Risk Factor Dermatology, Cohort Study |
| 130. Jung, J. H., McLaughlin, J. L., Stannard, J. & Guin, J. D. Isolation, via activity-directed fractionation, of mercaptobenzothiazole and dibenzothiazyl disulfide as 2 allergens responsible for tennis shoe dermatitis. Contact Dermatitis 19, 254–259 (1988). | Case Study | Not Reported in Abstract | Effects of Footwear, Risk Factor Injury/Pathology, Risk Factor Dermatology, Footwear Design |
| 131. Kanatlı, U. et al. Do corrective shoes improve the development of the medial longitudinal arch in children with flexible flat feet? J. Orthop. Sci. 21, 662–666 (2016). | Non Randomised Control Design | Not Reported in Abstract | Anthropometrics, Developmental Effects, Therapeutic Footwear, Therapeutic Footwear Corrective, Effects of Footwear |
| 132. Kato, T. & Watanabe, S. The etiology of Hallux Valgus in Japan. Clin Orthop Relat Res No.157, 78–81 (1981) | Opinion Piece | Not Applicable | Developmental Effects, Footwear Design |
| 133. Kayani, B. et al. Orthopaedic manifestations of congenital indifference to pain with anhidrosis (Hereditary Sensory and Autonomic Neuropathy type IV). Eur. J. Paediatr. Neurol. EJPN Off. J. Eur. Paediatr. Neurol. Soc. 21, 318–326 (2017). | Case-Series | Preschool and Infants 9mths-5yrs, Primary School 6-12yrs | Therapeutic Footwear, Therapeutic Footwear Functional, Therapeutic Footwear Functional Lift, Effects of Footwear |
| 134. Kebede, S. W., Beyene, D. A., Meshesha, A. G. & Sinishaw, M. A. Two thirds of hookworm infected children were anemic at the outpatient department in Jimma Health Center, Jimma, Southwest Ethiopia. Asian Pacific J. Trop. Dis. 6, 691–694 (2016). | Cross Sectional Study | Not Reported in Abstract | Effects of Footwear, Protective Role, Protective Role Infective |
| 135. Ketema, H., Biruksew, A. & Mekonnen, Z. Prevalence of Necator americanus infection and risk factors among school-age children in Mirab Abaya District, South Ethiopia. Asian Pacific J. Trop. Dis. 5, 363–368 (2015). | Cross Sectional Study | Not Reported in Abstract | Effects of Footwear, Protective Role, Protective Role Infective |
| 136. Khan, M. Y. An analytical study of factors related to infestation by intestinal parasites in rural school children (report of a pilot study). Public Health 93, 82–88 (1979). | Cross Sectional Study | Not Reported in Abstract | Effects of Footwear, Protective Role, Protective Role Infective |
| 137. Khieu, V. et al. Strongyloides stercoralis infection and re-infection in a cohort of children in Cambodia. Parasitol. Int. 63, 708–712 (2014). | Cross Sectional Study | Primary School 6-12yrs | Effects of Footwear, Protective Role, Protective Role Infective |
| 138. Klein, C., Groll-Knapp, E., Kundi, M. & Kinz, W. Increased hallux angle in children and its association with insufficient length of footwear: a community based cross-sectional study. BMC Musculoskelet Disord 10, 159 (2009). | Cross Sectional Study | Preschool and Infants 9mths-5yrs | Anthropometrics, Developmental Effects, Footwear Design, Effects of Footwear |
| 139. Knittel, G. & Staheli, L. T. The effectiveness of shoe modifications for intoeing. Orthop. Clin. North Am. 7, 1019–1025 (1976). | Before-and-after study | Not Reported in Abstract | Biomechanics, Developmental Effects, Effects of Footwear, Therapeutic Footwear, Therapeutic Footwear Functional, Therapeutic Footwear Functional Stability, Footwear Design |
| 140. Koch, P. & Nickolaus, G. Allergic contact dermatitis and mercury exanthem due to mercury chloride in plastic boots. Contact Dermatitis 34, 405–409 (1996). | Case Study | 5 years | Effects of Footwear, Risk Factor Injury/Pathology, Risk Factor Dermatology, Footwear Design |
| 141. Kolsek, T., Jurca, A. & Vidic, T. Survey on parents’ selection of children’s footwear. Footwear Sci. 3, S88–S90 (2011). | Survey | Not Reported in Abstract | Anthropometrics, Footwear Design, Psychosocial |
| 142. Kraemer, J. Loop sandals for early functional treatment of hallux valgus. Orthopadische Prax. 16, 882–884 (1980). | Opinion Piece | Not Applicable | Therapeutic Footwear, Therapeutic Footwear Corrective, Footwear Design, Effects of Footwear, Developmental Effects |
| 143. Kristen, H. Wholesome shoes for children. The role of the doctor in this problem. Osterr. Arzteztg. 29, 1299–1314 (1974). | Opinion Piece | Not Applicable | Developmental Effects, Footwear Design |
| 144. Kristen, K. H. et al. [Functional evaluation of shoes for children based on gait analysis of children in the learning to walk stage]. Z Orthop Ihre Grenzgeb 136, 457–462 (1998). | Before-and-after study | Preschool and Infants 9mths-5yrs | Biomechanics, Developmental Effects, Effects of Footwear, Footwear Design |
| 145. Krol, R. Treatment of deformities associated with congenital absence of the fibula. Chir Narzadow Ruchu Ortop Pol 45, 161–165 (1980). | Opinion Piece | Not Applicable | Therapeutic Footwear, Therapeutic Footwear Corrective, Footwear Design |
| 146. Kung, S. M., Fink, P. W., Hume, P. & Shultz, S. P. Kinematic and kinetic differences between barefoot and shod walking in children. Footwear Sci. 7, 95–105 (2015). | Before-and-after study | Primary School 6-12yrs | Biomechanics, Developmental Effects, Effects of Footwear, Cross Sectional Study |
| 147. Küper, K. et al. Evaluation of inner shoe dimensions by computed tomography. Fuss und Sprunggelenk 3, 159–163 (2005). | Cross Sectional Study | Not Reported in Abstract | Anthropometrics, Developmental Effects, Footwear Design |
| 148. Kurup, H. V, Clark, C. I. M. & Dega, R. K. Footwear and orthopaedics. Foot Ankle Surg 18, 79–83 (2012). | Narrative Review | Not Reported in Abstract | Developmental Effects, Footwear Design, Effects of Footwear |
| 149. Lachapelle, J. M. & Tennstedt, D. Juvenile plantar dermatosis: a report of 80 cases. Am J Ind Med 8, 291–295 (1985). | Case Series | Not Reported in Abstract | Effects of Footwear, Risk Factor Injury/Pathology, Risk Factor Dermatology, Footwear Design |
| 150. Lago, D. C. F., Coutinho, C. A., Kochi, C. & Longui, C. A. Reported shoes size during gh therapy: Is foot overgrowth a myth or reality? Arch Endocrinol Metab 59, 414–421 (2015). | Cross Sectional Study | Adolescent 13-18yrs | Anthropometrics, Footwear Design |
| 151. Lam, W. H. O., Lui, T. H. & Chan, K. M. The Epidemiology of Ankle Sprain During Hiking in Uniformed Groups. J. Orthop. Trauma Rehabil. 15, 10–16 (2011). | Cross Sectional Study | Adolescent 13-18yrs | Effects of Footwear, Risk Factor Injury/Pathology, Risk Factor Injury, Footwear Design |
| 152. Lampe, R., Mitternacht, J., Gerdesmeyer, L. & Gradinger, R. Plantar pressure measurement in children and youths during sports activities. Klin Padiatr 217, 70–75 (2005). | Cross Sectional Study | Adolescent 13-18yrs, Primary School 6-12yrs | Anthropometrics, Biomechanics, Effects of Footwear, Footwear Design |
| 153. Lampe, R. et al. Influence of orthopaedic-technical aid on the kinematics and kinetics of the knee joint of patients with neuro-orthopaedic diseases. Brain Dev. 26, 219–226 (2004). | Before-and-after study | Not Reported in Abstract | Biomechanics, Developmental Effects, Effects of Footwear, Therapeutic Footwear, Therapeutic Footwear Functional, Therapeutic Footwear Functional Stability |
| 154. Latorre Roman, P. A., Balboa, F. R. & Pinillos, F. G. Foot strike pattern in children during shod-unshod running. Gait Posture 58, 220–222 (2017). | Before-and-after study | Adolescent 13-18yrs, Primary School 6-12yrs | Developmental Effects, Biomechanics, Effects of Footwear |
| 155. Leong Lim, K. B. et al. Escalators, rubber clogs, and severe foot injuries in children. J Pediatr Orthop 30, 414–419 (2010). | Cross Sectional Study | Preschool and Infants 9mths-5yrs, Primary School 6-12yrs | Effects of Footwear, Risk Factor Injury/Pathology, Risk Factor Injury, Footwear Design |
| 156. Li, H., Zhao, C., Zhou, J., Shao, H. & Chen, W. Isolation, purification and identification of bacteria from the shoes worn by children. African J. Biotechnol. 10, 4133–4137 (2011). | Cross Sectional Study | Primary School 6-12yrs | Risk Factor Injury/Pathology, Risk Factor Infective, Footwear Design, Cross Sectional Study |
| 157. Li, H., Zhou, J., Shi, R. & Chen, W. Identification of fungi from children’s shoes and application of a novel antimicrobial agent on shoe insole. African J. Biotechnol. 10, 14493–14497 (2011). | Cross Sectional Study | Preschool and Infants 9mths-5yrs, Primary School 6-12yrs | Effects of Footwear, Risk Factor Injury/Pathology, Risk Factor Infective |
| 158. Li, Y. H. & Leong, J. C. Intoeing gait in children. Hong Kong Med. J. = Xianggang Yi Xue Za Zhi 5, 360–366 (1999). | Narrative Review | Not Reported in Abstract | Developmental Effects, Therapeutic Footwear, Therapeutic Footwear Corrective, Effects of Footwear |
| 159. Lieberman, D. E. et al. Foot strike patterns and collision forces in habitually barefoot versus shod runners. Nature 463, 531–535 (2010). | Cross Sectional Study | Not Reported in Abstract | Biomechanics, Developmental Effects, Effects of Footwear |
| 160. Lim, P. Q. X. et al. The association of foot structure and footwear fit with disability in children and adolescents with Down syndrome. J Foot Ankle Res 8, 4 (2015). | Cross Sectional Study | Adolescent 13-18yrs, Primary School 6-12yrs | Anthropometrics, Developmental Effects, Footwear Design, Effects of Footwear |
| 161. Lim, P. et al. Do foot posture, deformity, and footwear fit influence physical activity levels in children with Down syndrome? A prospective cohort study. J. Intellect. Dev. Disabil. 42, 332–338 (2017). | Cross Sectional Study | Primary School 6-12yrs | Anthropometrics, Developmental Effects, Footwear Design, Effects of Footwear |
| 162. López Elvira, J. L., López Plaza, D., López Valenciano, A. & Alonso Montero, C. Influencia del calzado en el movimiento del pie durante la marcha y la carrera en niños y niñas de 6 y 7 años. / Influence of footwear on foot movement during walking and running in boys and girls aged 6-7. Retos Nuevas Perspect. Educ. Física, Deport. y Recreación 31, 128–132 (2017). | Before-and-after study | Primary School 6-12yrs | Biomechanics, Developmental Effects, Effects of Footwear, Footwear Design |
| 163. Lord, M. & Foulston, J. Clinical trial of a computer-aided system for orthopaedic shoe upper design. Prosthet Orthot Int 15, 11–17 (1991). | Cross Sectional Study | Not Reported in Abstract | Footwear Design, Therapeutic Footwear |
| 164. Lord, M., Foulston, J. & Smith, P. J. Technical Evaluation of a Cad System for Orthopaedic Shoe-Upper Design. Proc. Inst. Mech. Eng. Part H J. Eng. Med. 205, 109–115 (1991). | Cross Sectional Study | Not Reported in Abstract | Footwear Design, Therapeutic Footwear |
| 165. Louey, M. G. Y. & Sangeux, M. Shod wear and foot alignment in clinical gait analysis. Gait Posture 49, 144–147 (2016). | Before-and-after study | Primary School 6-12yrs | Biomechanics, Effects of Footwear |
| 166. Lythgo, N., Wilson, C. & Galea, M. Basic gait and symmetry measures for primary school-aged children and young adults whilst walking barefoot and with shoes. Gait Posture 30, 502–506 (2009). | Before-and-after study | Primary School 6-12yrs | Biomechanics, Developmental Effects, Effects of Footwear |
| 167. Mackie, R. M. & Husain, S. L. Juvenile plantar dermatosis: A new entity? Clin Exp Dermatol 1, 253–260 (1976). | Case Series | Not Reported in Abstract | Effects of Footwear, Risk Factor Injury/Pathology, Risk Factor Dermatology |
| 168. Makary, M. A. Reported incidence of injuries caused by street glass among urban children in Philadelphia. Inj. Prev. 4, 148–149 (1998). | Cross Sectional Study | Not Reported in Abstract | Effects of Footwear, Protective Role, Protective Role Environmental |
| 169. Malas, B. S. What variables influence the ability of an AFO to improve function and when are they indicated? Clin Orthop Relat Res 469, 1308–1314 (2011). | Narrative Review | Not Reported in Abstract | Therapeutic Footwear, Therapeutic Footwear Functional, Therapeutic Footwear Functional Stability, Effects of Footwear |
| 170. Martin-Casado, L., Barquin, C., Martín-Casado, L. & Barquín, C. Does a Physical Education lesson affect the foot morphology in school-aged children? Bol. Med. Hosp. Infant. Mex. 74, 357–363 (2017). | Cross Sectional Study | Primary School 6-12yrs | Anthropometrics, Footwear Design |
| 171. Matsuda, S., Kasuga, K., Hanai, T., Demura, T. & Komura, K. The effect of the kindergarten barefoot policy on Preschool children’s toes. J Physiol Anthr. 36, 4 (2016). | Cross Sectional Study | Preschool and Infants 9mths-5yrs | Anthropometrics, Developmental Effects, Effects of Footwear |
| 172. Mauch, M., Grau, S., Krauss, I., Maiwald, C. & Horstmann, T. A new approach to children’s footwear based on foot type classification. Ergonomics 52, 999–1008 (2009). | Cross Sectional Study | Not Reported in Abstract | Anthropometrics, Footwear Design |
| 173. Mauch, M. et al. Do the feet of German and Australian children differ in structure? Implications for children’s shoe design. Ergonomics 51, 527–539 (2008). | Cross Sectional Study | Preschool and Infants 9mths-5yrs, Primary School 6-12yrs | Anthropometrics, Footwear Design |
| 174. McCarthy, C, et al. The effects of shoe design on gait, stability, and loading in new walkers, Abstracts * of Poster and Platform Presentations at the 2011. Pediatr. Phys. Ther. 23, 49–68 (2011). | Before-and-after study | Preschool and Infants 9mths-5yrs | Biomechanics, Developmental Effects, Effects of Footwear, Footwear Design |
| 175. McSweeney, S. C., Reed, L. F. & Wearing, S. The effect of sex on measures of foot mobility and stiffness in children and adolescents. Footwear Sci. 9, S138–S139 (2017). | Cross Sectional Study | Primary School 6-12yrs,  Adolescent 13-18yrs | Anthropometrics, Footwear Design |
| 176. Mendelewich, I. A. & Pitkin, M. R. Orthopedic shoes for children with cerebral palsy in residual stage and with ‘pes equino-varus congenital’ which is feebly marked or was surgically treated. Abstr. XII Congr. Int. Soc. Biomech. 22, 1055 (1989). | Opinion Piece | Not Applicable | Therapeutic Footwear, Therapeutic Footwear Functional, Therapeutic Footwear Functional Stability, Footwear Design |
| 177. Minkin, W., Cohen, H. J. & Frank, S. B. Contact Dermatitis to East Indian Leather. Arch Dermatol 103, 522–523 (1971). | Case Study | Not Reported in Abstract | Effects of Footwear, Risk Factor Injury/Pathology, Risk Factor Dermatology, Footwear Design |
| 178. MJ, I. J., Nene, A. V, Ijzerman, M. J. & Nene, A. V. Feasibility of the physiological cost index as an outcome measure for the assessment of energy expenditure during walking. Arch. Phys. Med. Rehabil. 83, 1777–1782 (2002). | Before-and-after study | Not Reported in Abstract | Physiological, Effects of Footwear |
| 179. Molla, Y. B. et al. Individual correlates of podoconiosis in areas of varying endemicity: a case-control study. PLoS Negl Trop Dis 7, e2554–e2554 (2013). | Cross Sectional Study | Not Reported in Abstract | Effects of Footwear, Protective Role, Protective Role Environmental |
| 180. Monsia, A. et al. [Surgery of congenital clubfoot in Don Orione, Health Center for physical handicaps of Ivory Coast (About 554 feet)]. Ann Chir Plast Esthet 53, 41–45 (2008). | Cross Sectional Study | Adolescent 13-18yrs, Preschool and Infants 9mths-5yrs, Primary School 6-12yrs | Anthropometrics, Developmental Effects, Therapeutic Footwear, Therapeutic Footwear Corrective, Effects of Footwear |
| 181. Moorthy, T. T. & Rajan, V. S. Juvenile plantar dermatosis in Singapore. Int J Dermatol 23, 476–479 (1984). | Cross Sectional Study | Adolescent 13-18yrs, Preschool and Infants 9mths-5yrs, Primary School 6-12yrs | Effects of Footwear, Risk Factor Injury/Pathology, Risk Factor Dermatology Footwear Design |
| 182. Moreno-Hernández, A. et al. Temporal and spatial gait parameters analysis in non-pathological Mexican children. Gait Posture 32, 78–81 (2010). | Before-and-after study | Primary School 6-12yrs | Biomechanics, Developmental Effects, Effects of Footwear |
| 183. Mueller, R. & Boltze, W. H. The conservative equalization of differences in the length of the legs. Ther. Umschau 32, 303–305 (1975). | Opinion Piece | Not Applicable | Therapeutic Footwear, Therapeutic Footwear Functional, Therapeutic Footwear Functional Lift, Footwear Design |
| 184. Mullen, S. & Toby, E. B. Adolescent runners: The effect of training shoes on running kinematics. J. Pediatr. Orthop. 33, 453–457 (2013). | Before-and-after study | Adolescent 13-18yrs | Biomechanics, Developmental Effects, Effects of Footwear, Footwear Design |
| 185. Muller, S. et al. Conservative treatment measures in hemophilic arthropathy. Orthopade 28, 347–355 (1999). | Case Series | Not Reported in Abstract | Therapeutic Footwear, Therapeutic Footwear Functional, Therapeutic Footwear Functional Stability, Effects of Footwear |
| 186. Muñoz-Antoli, C., Pavón, A., Marcilla, A., Toledo, R. & Esteban, J. G. Prevalence and risk factors related to intestinal parasites among children in Department of Rio San Juan, Nicaragua. Trans R Soc Trop Med Hyg 108, 774–782 (2014). | Cross Sectional Study | Adolescent 13-18yrs, Preschool and Infants 9mths-5yrs, Primary School 6-12yrs | Effects of Footwear, Protective Role, Protective Role Infective |
| 187. Munuera, P. V, Castillo, J. M., Dominguez, G. & Lafuente, G. Orthotic devices with out-toeing wedge as treatment for in-toed gait in children. J Am Pod. Med Assoc 100, 472–478 (2010). | Before-and-after study | Adolescent 13-18yrs, Preschool and Infants 9mths-5yrs, Primary School 6-12yrs | Biomechanics, Developmental Effects, Effects of Footwear |
| 188. Murri, A. & Zechner, G. [Corrective dynamic shoe fitting of the functional clubfoot in patients with infantile cerebral palsy]. Z Orthop Ihre Grenzgeb 132, 214–220 (1994). | Cross Sectional Study | Not Reported in Abstract | Anthropometrics, Developmental Effects, Therapeutic Footwear, Therapeutic Footwear Corrective, Effects of Footwear |
| 189. Nasr, N. A., Al-Mekhlafi, H. M., Ahmed, A., Roslan, M. A. & Bulgiba, A. Towards an effective control programme of soil-transmitted helminth infections among Orang Asli in rural Malaysia. Part 2: Knowledge, attitude, and practices. Parasit. Vectors 6, 28 (2013). | Cross Sectional Study | Not Reported in Abstract | Effects of Footwear, Protective Role, Protective Role Infective |
| 190. Neering, H. & Van Dijk, E. Juvenile plantar dermatosis. Acta Derm Venereol 58, 531–534 (1978). | Narrative Review | Adolescent 13-18yrs, Preschool and Infants 9mths-5yrs, Primary School 6-12yrs | Effects of Footwear, Risk Factor Injury/Pathology, Risk Factor Dermatology |
| 191. Neto, H. P. et al. Immediate effect of postural insoles on gait performance of children with cerebral palsy: Preliminary randomized controlled double-blind clinical trial. J. Phys. Ther. Sci. 26, 1003–1007 (2014). | Before-and-after study | Primary School 6-12yrs | Biomechanics, Effects of Footwear |
| 192. Nordenfelt, P. J. Children’s shoes. Acta Pædiatrica 25, 220–226 (1939). | Opinion Piece | Not Applicable | Developmental Effects, Footwear Design |
| 193. Ocak, B. & Gülümser, G. Foot measurement standardization of adolescent boys in 7-14 age group. Tekst. ve Konfeksiyon 19, 157–162 (2009). | Cross Sectional Study | Adolescent 13-18yrs, Primary School 6-12yrs | Anthropometrics, Footwear Design |
| 194. Oeffinger, D. et al. Comparison of gait with and without shoes in children. Gait Posture 9, 95–100 (1999). | Before-and-after study | Not Reported in Abstract | Biomechanics, Developmental Effects, Effects of Footwear |
| 195. Ortiz-Salvador, J.-M. M. et al. Dermatitis of the Foot: Epidemiologic and Clinical Features in 389 Children. Pediatr Dermatol 34, 535–539 (2017). | Cross Sectional Study | Not Reported in Abstract | Effects of Footwear, Risk Factor Injury/Pathology, Risk Factor Dermatology, Footwear Design |
| 196. Pandey, S. et al. Flatfoot in Indian population. J. Orthop. Surg. (Hong Kong) 21, 32–36 (2013). | Randomised control Trial | Not Reported in Abstract | Anthropometrics, Developmental Effects, Therapeutic Footwear, Therapeutic Footwear Corrective, Effects of Footwear, Footwear Design |
| 197. Pasin Neto, H. et al. Postural insoles on gait in children with cerebral palsy: Randomized controlled double-blind clinical trial. J. Bodyw. Mov. Ther. 21, 890–895 (2017). | Randomised Control Trial | Primary School 6-12yrs | Biomechanics, Effects of Footwear |
| 198. Pauk, J. & Griškevičius, J. Ground reaction force and support moment in typical and flat-feet children. Mechanika 17, 93–96 (2011). | Cross Sectional Study | Not Reported in Abstract | Biomechanics, Effects of Footwear, Footwear Design |
| 199. Pauk, J., Ezerskiy, V., Raso, J. V & Rogalski, M. Epidemiologic factors affecting plantar arch development in children with flat feet. J Am Pod. Med Assoc 102, 114–121 (2012). | Cross Sectional Study | Adolescent 13-18yrs Primary School 6-12yrs | Anthropometrics, Developmental Effects, Effects of Footwear |
| 200. Pavlackova, J., Egner, P., Mokrejs, P. & Cernekova, M. Verification of toe allowance of children’s footwear and its categorisation. Footwear Sci. 7, 149–157 (2015). | Cross Sectional Study | Not Reported in Abstract | Developmental Effects, Footwear Design |
| 201. Penneau, K., Lutter, L. D. & Winter, R. D. Pes planus: radiographic changes with foot orthoses and shoes. Foot Ankle 2, 299–303 (1982). | Before-and-after study | Not Reported in Abstract | Anthropometrics, Therapeutic Footwear, Therapeutic Footwear Functional, Therapeutic Footwear Functional Stability, Effects of Footwear |
| 202. Pezzan, P. A. O., Sacco, I. C. N. & João, S. M. A. Foot posture and classification of the plantar arch among adolescent wearers and non-wearers of high-heeled shoes. Brazilian J. Phys. Ther. / Rev. Bras. Fisioter. 13, 398–404 (2009). | Cross Sectional Study | Adolescent 13-18yrs | Anthropometrics, Developmental Effects, Footwear Design, Effects of Footwear |
| 203. Phiri, K. S. The prevalence, intensity and ecological determinants of helminth infection among children in an urban and rural community in Southern Malawi. Malawi Med. J. J. Med. Assoc. Malawi 13, 22–26 (2001). | Cross Sectional Study | Adolescent 13-18yrs,  Preschool and Infants 9mths-5yrs, Primary School 6-12yrs | Effects of Footwear, Protective Role, Protective Role Infective |
| 204. Phiri, K., Whitty, C. J. M., Graham, S. M. & Ssembatya-Lule, G. Urban/rural differences in prevalence and risk factors for intestinal helminth infection in southern Malawi. Ann Trop Med Parasitol 94, 381–387 (2000). | Cross Sectional Study | Adolescent 13-18yrs,  Preschool and Infants 9mths-5yrs, Primary School 6-12yrs | Effects of Footwear, Protective Role, Protective Role Infective |
| 205. Prasher, V. P., Robinson, L., Krishnan, V. H. R. & Chung, M. C. Podiatric disorders among children with Down syndrome and learning disability. Dev Med Child Neurol 37, 131–134 (1995). | Cross Sectional Study | Adolescent 13-18yrs, Primary School 6-12yrs | Anthropometrics, Biomechanics, Effects of Footwear, Footwear Design |
| 206. Puszczałowska-Lizis, E. et al. Foot Structure in Boys with Down Syndrome. Biomed Res. Int. 2017, 7047468 (2017). | Cross Sectional Study | Adolescent 13-18yrs | Anthropometrics, Footwear Design |
| 207. Raczkowski, J. W., Daniszewska, B. & Zolynski, K. Functional scoliosis caused by leg length discrepancy. Arch. Med. Sci. 6, 393–398 (2010). | Cohort Study | Adolescent 13-18yrs, Primary School 6-12yrs | Anthropometrics, Developmental Effects, Therapeutic Footwear, Therapeutic Footwear Functional, Therapeutic Footwear Functional Lift, Effects of Footwear |
| 208. Rajchel-Chyla, B., Skrzyńska, B., Janocha, M. & Gajewski, R. The foot length changes due to age as well as load during ambulation and determintion of the toe allowance. Prz. Wlokienniczy 66, 23–26 (2012). | Cross Sectional Study | Not Reported in Abstract | Anthropometrics, Footwear Design, Footwear Design |
| 209. Ramstrand, N., Andersson, C. B., Rusaw, D., Björk Andersson, C. & Rusaw, D. Effects of an unstable shoe construction on standing balance in children with developmental disabilities: A pilot study. Prosthet Orthot Int 32, 422–433 (2008). | Pilot Study | Adolescent 13-18yrs,  Primary School 6-12yrs | Biomechanics, Developmental Effects, Effects of Footwear Therapeutic Footwear, Therapeutic Footwear Functional, Therapeutic Footwear Functional Unstable |
| 210. Reichert, F. et al. Prevalence and Risk Factors of Hookworm-Related Cutaneous Larva Migrans (HrCLM) in a Resource-Poor Community in Manaus, Brazil. PLoS Negl Trop Dis 10, e0004514–e0004514 (2016). | Cross Sectional Study | Adolescent 13-18yrs Primary School 6-12yrs | Effects of Footwear, Protective Role, Protective Role Infective |
| 211. Revenga-Giertych, C. & Bulo-Concellón, M. P. Valgus flatfoot: Evolution of the footprint and related factors. Rev. Ortop. y Traumatol. 49, 271–280 (2005). | Cross Sectional Study | Adolescent 13-18yrs, Primary School 6-12yrs | Anthropometrics, Developmental Effects,, Effects of Footwear |
| 212. Robinson, L. E. et al. Footwear and locomotor skill performance in Preschoolers. Percept Mot Ski. 113, 534–538 (2011). | Randomised Control Trail | Preschool and Infants 9mths-5yrs | Biomechanics, Developmental Effects, Effects of Footwear, Footwear Design |
| 213. Rocha, E. S. & Pedreira, A. C. [Common orthopedic problems in adolescents]. J. Pediatr. (Rio. J). 77 Suppl 2, S225–S233 (2001). | Narrative Review | Not Reported in Abstract | Developmental Effects, Therapeutic Footwear, Therapeutic Footwear Corrective, Effects of Footwear |
| 214. Romaguera, C. & Vilaplana, J. Contact dermatitis in children: 6 years experience (1992-1997). Contact Dermatitis 39, 277–280 (1998). | Cross Sectional Study | Adolescent 13-18yrs | Effects of Footwear, Risk Factor Injury/Pathology, Risk Factor Dermatology, Footwear Design |
| 215. Rome, K., Ashford, R. L. & Evans, A. Non-surgical interventions for paediatric pes planus. Cochrane Database Syst Rev 7, Cd006311 (2010). | Systematic Review | Preschool and Infants 9mths-5yrs, Primary School 6-12yrs | Developmental Effects, Therapeutic Footwear, Therapeutic Footwear Corrective, Effects of Footwear |
| 216. Rotter, I., Wicher, J., Zułtak-Ba̧czkowska, K., Mroczek, B. & Karakiewicz, B. Prevention and correction of faulty postures among children in pre-school age-parents’ opinion. Fam. Med. Prim. Care Rev. 11, 471–472 (2009). | Survey | Preschool and Infants 9mths-5yrs | Developmental Effects, Psychosocial, Therapeutic Footwear, Therapeutic Footwear Corrective, Effects of Footwear |
| 217. Roul, S. et al. Footwear contact dermatitis in children. Contact Dermatitis 35, 334–336 (1996). | Case Series | Preschool and Infants 9mths-5yrs, Primary School 6-12yrs | Effects of Footwear, Risk Factor Injury/Pathology, Risk Factor Dermatology |
| 218. Roye, D. P. J. & Raimondo, R. A. Surgical treatment of the child’s and adolescent’s flexible flatfoot. Clin. Podiatr. Med. Surg. 17, 515–30, vii–viii (2000). | Opinion Piece | Not Applicable | Therapeutic Footwear, Therapeutic Footwear Functional, Therapeutic Footwear Functional Stability, Footwear Design |
| 219. Sacco, I. C. N., Onodera, A. N., Bosch, K., Rosenbaum, D. & Sacco, I. C. N. Comparisons of foot anthropometry and plantar arch indices between German and Brazilian children. BMC Pediatr. 15, 4 (2015). | Cross Sectional Study | Preschool and Infants 9mths-5yrs, Primary School 6-12yrs | Anthropometrics, Footwear Design |
| 220. Sachithanandam, V. & Joseph, B. The influence of footwear on the prevalence of flat foot. A survey of 1846 skeletally mature persons. J. Bone Jt. Surg. - Ser. B 77, 254–257 (1995). | Cross Sectional Study | Adult Population Retrospective to Childhood | Anthropometrics, Developmental Effects, Effects of Footwear |
| 221. Sah, R. B. et al. Prevalence of intestinal helminthic infections and associated risk factors. Indian J. Community Heal. 25, 134–139 (2013). | Cross Sectional Study | Not Reported in Abstract | Effects of Footwear, Protective Role, Protective Role Infective |
| 222. Sandoval, N. R. et al. A survey of intestinal parasites including associated risk factors in humans in Panama. Acta Trop 147, 54–63 (2015). | Cross Sectional Study | Not Reported in Abstract | Effects of Footwear, Protective Role, Protective Role Infective |
| 223. Sanzarello, I., Nanni, M. & Faldini, C. The clubfoot over the centuries. J. Pediatr. Orthop. Part B 26, 143–151 (2017). | Narrative Review | Not Reported in Abstract | Developmental Effects, Therapeutic Footwear, Therapeutic Footwear Corrective, Effects of Footwear |
| 224. Sass, P. & Hassan, G. Lower extremity abnormalities in children. Am Fam Physician 68, 461–468 (2003). | Opinion Piece | Not Applicable | Developmental Effects, Therapeutic Footwear, Therapeutic Footwear Corrective, Footwear Effects |
| 225. Schaars, A. H. & Postema, K. Conservative treatment of foot deformities in Duchenne Muscular Dystrophy. J. Rehabil. Sci. 3, 49–52 (1990). | Opinion Piece | Not Applicable | Therapeutic Footwear, Therapeutic Footwear Functional, Therapeutic Footwear Functional Stability, Effects of Footwear |
| 226. Shin, I. S., Kim, E. S. & Kim, J. T. The segemental measurement of foot and its three dimensional modeling. Korean J. Sport Sci. 3, 48–65 (1991). | Cross Sectional Study | Adolescent 13-18yrs,  Primary School 6-12yrs | Anthropometrics, Footwear Design |
| 227. Shultz, S. P., Houltham, S. D., Kung, S. M., Hume, P. & Fink, P. W. Metabolic Differences between Shod and Barefoot Walking in Children. Int J Sport. Med 37, 401–404 (2016). | Before-and-after study | Primary School 6-12yrs | Developmental Effects, Physiological, Effects of Footwear |
| 228. Silva, A. M., de Siqueira, G. R. & da Silva, G. A. P. Implications of high-heeled shoes on body posture of adolescents. Rev. Paul. Pediatr. Orgao Of. Da Soc. Pediatr. Sao Paulo 31, 265–271 (2013). | Narrative Review | Adolescent 13-18yrs | Anthropometrics, Biomechanics, Developmental Effects, Effects of Footwear, Effects of Footwear, Footwear Design |
| 229. Simon, T. D., Soep, J. B. & Hollister, J. R. Pernio in pediatrics. Pediatrics 116, e472–e475 (2005). | Case Series | Not Reported in Abstract | Effects of Footwear, Risk Factor Injury/Pathology, Risk Factor Injury, Footwear Design |
| 230. Society, C. P. Footwear for children. Paediatr. Child Health 3, 373–375 (1998). | Opinion Piece | Not Applicable | Developmental Effects, Footwear Design |
| 231. Staheli, L. T. Torsional deformity. Pediatr Clin North Am 33, 1373–1383 (1986). | Opinion Piece | Not Applicable | Developmental Effects, Therapeutic Footwear, Therapeutic Footwear Corrective, Effects of Footwear |
| 232. Staheli, L. T. Planovalgus foot deformity. Current status. J Am Pod. Med Assoc 89, 94–99 (1999). | Opinion Piece | Not Applicable | Developmental Effects, Therapeutic Footwear, Therapeutic Footwear Corrective, Effects of Footwear |
| 233. Staheli, L. T. Corrective shoes for children: are they really necessary? J. Musculoskelet. Med. 13, 11–15 (1996). | Opinion Piece | Not Applicable | Developmental Effects, Therapeutic Footwear, Therapeutic Footwear Corrective, Effects of Footwear |
| 234. Staheli, L. T. Shoes for children: a review. Pediatrics 88, 371–375 (1991). | Narrative Review | Not Reported in Abstract | Developmental Effects Therapeutic Footwear, Therapeutic Footwear Corrective, Effects of Footwear |
| 235. Staheli, L. T. & Giffin, L. Corrective shoes for children: a survey of current practice. Pediatrics 65, 13–17 (1980). | Survey | Not Reported in Abstract | Developmental Effects, Psychosocial, Therapeutic Footwear, Therapeutic Footwear Corrective, Effects of Footwear |
| 236. Steen, H., Terjesen, T. & Bjerkreim, I. [Anisomelia. Clinical consequences and treatment]. Tidsskr. Den Nor. Laegeforening Tidsskr. Prakt. Med. Ny Raekke 117, 1595–1600 (1997). | Opinion Piece | Not Applicable | Therapeutic Footwear, Therapeutic Footwear Functional, Therapeutic Footwear Functional Lift, Footwear Design |
| 237. Stricker, S. J. & Sama, A. A. Assessment of angulation and torsion of lower limbs in children. Int. Pediatr. 16, 138–143 (2001). | Narrative Review | Not Reported in Abstract | Developmental Effects, Therapeutic Footwear, Therapeutic Footwear Corrective, Effects of Footwear |
| 238. Taeho, Y. Parent, peer and TV influences on American teens’ athletic shoes purchasing. Int. J. Sport Manag. Mark. 1, 2 (2005). | Survey | Adolescent 13-18yrs | Psychosocial, Footwear Effects |
| 239. Talusan, P. G., Milewski, M. D., Reach Jr, J. S. & Reach Jr., J. S. Fifth Toe Deformities: Overlapping and Underlapping Toe. Foot Ankle Spec. 6, 145–149 (2013). | Narrative Review | Not Reported in Abstract | Therapeutic Footwear, Therapeutic Footwear Accommodative, Footwear Design, Effects of Footwear |
| 240. Theophilos, P. et al. Evaluation of sprinting performance in adolescent athletes with running shoes, spikes and barefoot. J. Phys. Educ. Sport 14, 593–598 (2014). | Before-and-after study | Primary School 6-12yrs | Biomechanics, Effects of Footwear |
| 241. Thompson, A. L. T. & Zipfel, B. The unshod child into womanhood -- forefoot morphology in two populations. Foot 15, 22–28 (2005). | Cross Sectional Study | Not Reported in Abstract | Anthropometrics, Developmental Effects, Effects of Footwear |
| 242. Tomono, N. et al. Risk factors of helminthiases among schoolchildren in southern Thailand. Southeast Asian J Trop Med Public Heal. 34, 264–268 (2003). | Cross Sectional Study | Primary School 6-12yrs | Effects of Footwear, Protective Role, Protective Role Infective |
| 243. Tong, J. W. K., Pui, W. K., Kong, P. W., Pui, W. K. & Kong, P. W. Medial Longitudinal Arch Development of Children Aged 7 to 9 Years: Longitudinal Investigation. Phys Ther 96, 1216–1224 (2016). | Cross Sectional Study | Primary School 6-12yrs | Anthropometrics, Developmental Effects, Effects of Footwear |
| 244. Trevisan, G., Kokeli, E. & Kokelj, F. Allergic contact dermatitis due to shoes in children: a 5 year follow up. Contact Dermatitis 26, 45–45 (1992). | Cross Sectional Study | Adolescent 13-18yrs,  Primary School 6-12yr | Effects of Footwear, Risk Factor Injury/Pathology, Risk Factor Dermatology , Footwear Design |
| 245. Tulu, B., Taye, S. & Amsalu, E. Prevalence and its associated risk factors of intestinal parasitic infections among Yadot primary school children of South Eastern Ethiopia: A cross-sectional study. BMC Res. Notes 7, 848 (2014). | Cross Sectional Study | Primary School 6-12yrs | Effects of Footwear, Protective Role, Protective Role Infective |
| 246. Uden, H. & Kumar, S. Non-surgical management of a pediatric ‘intoed’gait pattern - a systematic review of the current best evidence. J. Multidiscip. Healthc. 5, 27–35 (2012). | Systematic Review | Not Reported in Abstract | Therapeutic Footwear, Therapeutic Footwear Functional, Therapeutic Footwear Functional Stability, Effects of Footwear, Developmental Effects |
| 247. Ujević, D. et al. Standardization, anthropometric surveys and croatian anthropometric system. Tekstil 55, 516–526 (2006). | Cross Sectional Study | Adolescent 13-18yrs  Primary School 6-12yrs | Anthropometrics, Footwear Design |
| 248. Unger, H. & Rosenbaum, D. Gender-specific differences of the foot during the first year of walking. Foot Ankle Int 25, 582–587 (2004). | Cross Sectional Study | Preschool and Infants 9mths-5yrs | Anthropometrics, Footwear Design Developmental Effects |
| 249. Uvelli, K., Neher, J. O. & Safranek, S. Treatment for Calcaneal Apophysitis. Am Fam Physician 96, 126–127 (2017). | Opinion Piece | Not Applicable | Effects of Footwear, Protective Role, Protective Role Functional |
| 250. Van Hamme, A. et al. Is there a predominant influence between heel height, upper height and sole stiffness on young children gait dynamics? Comput. Methods Biomech. Biomed. Engin. 16, 66–67 (2013). | Before-and-after study | Preschool and Infants 9mths-5yrs, Primary School 6-12yrs | Biomechanics, Developmental Effects, Effects of Footwear, Footwear Design |
| 251. Veilleux, L.-N. N., Ballaz, L., Robert, M., Lemay, M. & Rauch, F. Analysing gait using a force-measuring walkway: intrasession repeatability in healthy children and adolescents. Comput Methods Biomech Biomed Engin 17, 1447–1451 (2014). | Before-and-after study | Adolescent 13-18yrs, Primary School 6-12yrs | Biomechanics, Effects of Footwear |
| 252. Vogel Jr, F. Short-leg syndrome. Clin Pod. 1, 581–599 (1984). | Opinion Piece | Not Applicable | Therapeutic Footwear, Therapeutic Footwear Functional, Therapeutic Footwear Functional Lift, Footwear Design |
| 253. Vrdoljak, J. et al. Antropometric measurements of growing feet. Paediatr. Croat. 48, 117–120 (2004). | Cross Sectional Study | Adolescent 13-18yrs, Primary School 6-12yrs | Anthropometrics, Footwear Design |
| 254. Walker, S. L. et al. The prevalence and association with health-related quality of life of tungiasis and scabies in schoolchildren in southern Ethiopia. PLoS Negl Trop Dis 11, e0005808 (2017). | Cross Sectional Study | Not Reported in Abstract | Effects of Footwear, Protective Role, Protective Role Infective |
| 255. Walther Richters, M., Wahl, H., Walther-Richters, M. & Wahl, H. Problems in choice of shoes for young children. Beitr Orthop Traumatol 24, 181–184 (1977). | Survey | Preschool and Infants 9mths-5yrs | Developmental Effects, Footwear Design |
| 256. Walther, M. et al. Requirements for children sport shoes, when taking into consideration the evolution of the child’s foot. A systematic review of current literature. Fuss und Sprunggelenk 3, 23–33 (2005). | Systematic Review | Not Reported in Abstract | Developmental Effects, Footwear Design |
| 257. Walther, M., Herold, D., Sinderhauf, A. & Morrison, R. Children sport shoes--a systematic review of current literature. Foot Ankle Surg 14, 180–189 (2008). | Systematic Review | Not Reported in Abstract | Developmental Effects, Footwear Design |
| 258. Watanabe, E. et al. Use of footwear and foot condition among rural Ethiopian school children. J Epidemiol Glob Heal. 4, 323–325 (2014). | Cross Sectional Study | Primary School 6-12yrs | Effects of Footwear, Protective Role, Protective Role Environmental |
| 259. Wegener, C. et al. Effect of sports shoes on midfoot power generation in children while walking and running. Footwear Sci. 5, S55–S56 (2013). | Before-and-after study | Primary School 6-12yrs | Biomechanics, Developmental Effects, Effects of Footwear |
| 260. Wegener, C. et al. Effect of sports shoes on children’s vertical jump performance and midfoot and ankle kinetics. Footwear Sci. 5, S58–S59 (2013). | Before-and-after study | Primary School 6-12yrs | Biomechanics, Developmental Effects, Effects of Footwear |
| 261. Wegener, C. et al. In-shoe multi-segment foot kinematics of children during the propulsive phase of walking and running. Hum Mov Sci 39, 200–211 (2015). | Before-and-after study | Not Reported in Abstract | Biomechanics, Developmental Effects, Effects of Footwear |
| 262. Wegener, C. et al. Power generation of the midfoot in children wearing sports shoes. J. Foot Ankle Res. 2013 61 6, O35 (2013). | Before-and-after study | Not Reported in Abstract | Biomechanics, Developmental Effects, Effects of Footwear |
| 263. Wegener, C., Hunt, A. E., Vanwanseele, B., Burns, J. & Smith, R. M. Effect of children’s shoes on gait: a systematic review and meta-analysis. J Foot Ankle Res 4, 3 (2011). | Systematic Review | Adolescent 13-18yrs, Preschool and Infants 9mths-5yrs, Primary School 6-12yrs | Biomechanics, Developmental Effects, Effects of Footwear |
| 264. Wegener, C. et al. Three-dimensional ankle kinematics in children’s school shoes during running. J. Foot Ankle Res. 5, O20 (2012). | Before-and-after study | Primary School 6-12yrs | Biomechanics, Effects of Footwear, Footwear Design |
| 265. Wegener, C. et al. Children’s rearfoot and midfoot motion while walking in school shoes. J. Foot Ankle Res. 4, O49 (2011). | Before-and-after study | Primary School 6-12yrs | Biomechanics, Developmental Effects, Effects of Footwear |
| 266. Weiss, J., De Jong, A., Packer, E. & Bonanni, L. Purchasing infant shoes: attitudes of parents, pediatricians, and store managers. Pediatrics 67, 718–720 (1981). | Survey | Preschool and Infants 9mths-5yrs | Psychosocial, Developmental Effects, Footwear Design |
| 267. Wenger, D. R. & Leach, J. Foot deformities in infants and children. Pediatr Clin North Am 33, 1411–1427 (1986). | Opinion Piece | Not Applicable | Developmental Effects, Therapeutic Footwear, Therapeutic Footwear Corrective, Effects of Footwear |
| 268. Wenger, D. R. et al. Foot growth rate in children age one to six years. Foot Ankle Int 3, 207–210 (1983). | Cross Sectional Study | Preschool and Infants 9mths-5yrs | Anthropometrics, Footwear Design |
| 269. Wenger, D. R., Mauldin, D., Speck, G., Morgan, D. & Lieber, R. L. Corrective Shoes and Inserts as Treatment for Flexible Flatfoot in Infants and Children. J Bone Jt. Surg Am 71, 953–954 (1989). | Randomised Control Trial | Not Reported in Abstract | Anthropometrics, Developmental Effects, Therapeutic Footwear, Therapeutic Footwear Corrective, Effects of Footwear |
| 270. Weston, J. A., Hawkins, K. & Weston, W. L. Foot dermatitis in children. Pediatrics 72, 824–827 (1983). | Cross Sectional Study | Not Reported in Abstract | Effects of Footwear, Risk Factor Injury/Pathology, Footwear Design, Risk Factor Dermatology |
| 271. Williams, C. M., James, A. M. & Tran, T. Metatarsus adductus: development of a non-surgical treatment pathway. J Paediatr Child Heal. 49, E428-33 (2013). | Narrative Review | Not Reported in Abstract | Developmental Effects, Therapeutic Footwear, Therapeutic Footwear Corrective, Effects of Footwear |
| 272. Williams, C. M., Michalitsis, J., Murphy, A., Rawicki, B. & Haines, T. P. Do external stimuli impact the gait of children with idiopathic toe walking? A study protocol for a within-subject randomised control trial. BMJ Open 3, (2013). | Research Protocol | Not Reported in Abstract | Biomechanics, Developmental Effects, Effects of Footwear |
| 273. Williams, C. M., Tinley, P. & Rawicki, B. Idiopathic toe-walking: have we progressed in our knowledge of the causality and treatment of this gait type? J Am Pod. Med Assoc 104, 253–262 (2014). | Narrative Review | Not Reported in Abstract | Therapeutic Footwear, Therapeutic Footwear Functional, Therapeutic Footwear Functional Stability, Effects of Footwear |
| 274. Wolf, S. et al. Foot motion in children shoes: a comparison of barefoot walking with shod walking in conventional and flexible shoes. Gait Posture 27, 51–59 (2008). | Before-and-after study | Primary School 6-12yrs | Biomechanics, Developmental Effects, Effects of Footwear |
| 275. Xu, M. & Wang, L. [Foot growth and foot types in children and adolescents: a narrative review]. Sheng Wu Yi Xue Gong Cheng Xue Za Zhi 34, 648–652 (2017). | Narrative Review | Not Reported in Abstract | Anthropometrics, Developmental Effects, Footwear Design |
| 276. Yamamoto, A. A Study of the Foot Form for Footwear Design Part 8 :Factor Analysis of the Toes of Male and Female Children of 3 to 6 years old. J. JAPAN Res. Assoc. Text. END-USES 34, 254–260 (1993). | Cross Sectional Study | Preschool and Infants 9mths-5yrs | Anthropometrics, Footwear Design |
| 277. Yamamoto, A. & Imamatsu, R. A Study of the Foot Form for Footwear Design (Part1) —The Property of the Foot Form of Children Ranging from Three to Six in Age on the Basis of the Footprints or the Foot Measurements—. J. JAPAN Res. Assoc. Text. END-USES 31, 231–237 (1990). | Cross Sectional Study | Preschool and Infants 9mths-5yrs | Anthropometrics, Footwear Design |
| 278. Yamamoto, A. & Imamatsu, R. A Study of the Foot Form for Footwear Design (Part 2) —The Increment of Foot Growth of Children Aged Three to Six Traced and Measured—. J. JAPAN Res. Assoc. Text. END-USES 31, 245–249 (1990). | Cross Sectional Study | Preschool and Infants 9mths-5yrs | Anthropometrics, Footwear Design |
| 279. Yamamoto, S. Studies on upright postural sway in normal and cerebral palsy children. Acta Sch. Med. Univ. Gifu 33, 822–849 (1985). | Cross Sectional Study | Not Reported in Abstract | Biomechanics, Developmental Effects, Effects of Footwear |
| 280. Yi, N. et al. Improved influence of the combination of wedged shoes and ankle foot orthoses on the extension ability of knee joint in cerebral palsy children. J. Clin. Rehabil. Tissue Eng. Res. 11, 900–902 (2007). | Before-and-after study | Not Reported in Abstract | Biomechanics, Effects of Footwear,, Therapeutic Footwear, Therapeutic Footwear Functional, Therapeutic Footwear Functional Stability, Developmental Effects |
| 281. Yori, P. P. et al. Seroepidemiology of strongyloidiasis in the Peruvian Amazon. Am J Trop Med Hyg 74, 97–102 (2006). | Cross Sectional Study | Not Reported in Abstract | Effects of Footwear, Protective Role, Protective Role Infective |
| 282. Young, E. Forefoot eczema—further studies and a review. Clin Exp Dermatol 11, 523–528 (1986). | Case Series | Not Reported in Abstract | Effects of Footwear, Risk Factor Injury/Pathology, Risk Factor Dermatology |
| 283. Yurt, Y., Sener, G. & Yakut, Y. Footwear suitability in Turkish Preschool-aged children. Prosthetics Orthot. Int. (Taylor Fr. Ltd) 38, 224–231 (2014). | Cross Sectional Study | Preschool and Infants 9mths-5yrs | Anthropometrics, Developmental Effects, Footwear Design, Psychosocial |
| 284. Zabjek, K. F. et al. Acute postural adaptations induced by a shoe lift in idiopathic scoliosis patients. Eur. Spine J. Off. Publ. Eur. Spine Soc. Eur. Spinal Deform. Soc. Eur. Sect. Cerv. Spine Res. Soc. 10, 107–113 (2001). | Before-and-after study | Adolescent 13-18yrs Primary School 6-12yrs | Anthropometrics, Therapeutic Footwear, Therapeutic Footwear Functional, Therapeutic Footwear Functional Lift,  Effects of Footwear |
| 285. Zhou, J., Li, T., Xu, B. & Chen, W. Investigation of children’s plantar pressure distribution with varied angle of hallux. Leather Footwear J. 15, 3–14 (2015). | Cross Sectional Study | Preschool and Infants 9mths-5yrs, Primary School 6-12yrs | Biomechanics, Developmental Effects, Effects of Footwear, Footwear Design |
| 286. Žukiené, K., Vilunaité, L. & Milašiené, D. Analysis of Preschool age Lithuania children’s feet measurements: Implications for shoe design. in 4th International Textile, Clothing and Design Conference - Magic World of Textiles, ITC and DC 1047–1051 (2008). | Cross Sectional Study | Preschool and Infants 9mths-5yrs | Anthropometrics, Footwear Design |
| 287. What requirements to make of a well made child’s shoe? (Dutch). Ned. Tijdschr. voor Fysiother. 86, 117–119 (1976). | Opinion Piece | Not Applicable | Developmental Effects, Footwear Design |
